# Shizao decoction for cirrhotic ascites: assessing potential targets based on network analysis combined with pharmacokinetics and metabolomics

**DOI:** 10.3389/fphar.2024.1298818

**Published:** 2024-01-23

**Authors:** Wenjing Li, Yujiao Hou, Yanping Wang, Ronghong Liu, Han Zhang, Yanqiong Luo, Qian Li, Mosesmanaanye Njolibimi, Bo Hong, Tao Xu

**Affiliations:** ^1^ School of Pharmacy, Qiqihar Medical University, Qiqihar, China; ^2^ Comprehensive Support Center, Arongqi Medical Security Bureau, Hulunbuir, China; ^3^ Pharmacy Department, Xichong Traditional Chinese Medicine Hospital, Nanchong, China

**Keywords:** Shizao decoction, pharmacokinetics, metabolomic, network analysis, cirrhotic ascites

## Abstract

**Introduction:** Shizao decoction (SZD) is a traditional Chinese medicine decoction that has therapeutic effects on cirrhotic ascites (CAS). Because of the unclear treatment mechanism, in the current study, the anti-CAS activity of SZD and molecular mechanisms were analyzed by network analysis combined with pharmacokinetics and metabolomics.

**Methods:** Firstly, we assessed the anti-CAS efficacy of SZD by hematoxylin-eosin (H&E), liver function tests, NO and ET-1 levels, and portal venous pressure. Secondly, network analysis was applied to dig out the metabolites, targets, and pathways related to SZD and CAS. Then, the pharmacokinetics of the pharmacokinetically relevant metabolites (PRM) were analyzed. Thirdly, the serum and urine metabolic biomarkers of rats with CAS were identified using metabolomics by comparing them with the SZD treatment group. In addition, MetaboAnalyst was utilized to conduct metabolic pathway analysis. Finally, the correlation analysis established a dynamic connection between absorbed PRM from SZD and CAS-associated endogenous metabolites.

**Results:** Pharmacodynamic analysis indicated that SZD effectively mitigated liver injury symptoms by ameliorating inflammatory cell infiltration in CAS rats. The network analysis results indicated that twelve RPM contribute to the therapeutic efficacy of SZD against CAS; the key signaling pathways involved might be hepatitis B and PI3K-Akt. Pharmacokinetics results showed that the 12 RPM were efficiently absorbed into rat plasma, ensuring desirable bioavailability. The metabolomic analysis yielded 21 and 23 significantly distinct metabolites from the serum and urine, respectively. The 12 bioavailable SZD-PRM, such as luteolin, apigenin, and rutin, may be associated with various CAS-altered metabolites related to tryptophan metabolism, alpha-linolenic acid metabolism, glycine metabolism, etc.

**Discussion:** A novel paradigm was provided in this study to identify the potential mechanisms of pharmacological effects derived from a traditional Chinese medicine decoction.

## 1 Introduction

Chronic liver disease can irreversibly lead to cirrhosis, the most common and clinically relevant complication of cirrhosis (Gallo et al., 2020). Dysregulation of multiple intersecting biological pathways is intimately linked to the development of CAS. Current scientific strategies to discover therapeutic approaches for CAS usually focus on a single target: dehydration, so diuretic therapy is the standard traditional treatment method (Li et al., 2023). However, long-term use of diuretics can lead to endocrine disorders, diarrhea, and abdominal pain. Traditional Chinese Medicine (TCM) is multi-targeted and has been used clinically for thousands of years (Gao and Yao, 2019). Hence, exploring bioactive metabolites extracted from classical formulations of TCM with good therapeutic effects is a potentially effective way to discover novel therapeutics for CAS.

SZD, a very famous water-removing formula, is from “Treatise on Febrile Diseases” and has also been found in “Synopsis of the Golden Chamber” (Zhou et al., 2012). It comprises 4 botanical drugs: euphorbia (*Euphorbia pekinensis* Rupr.), flos genkwa (*Daphne genkwa* Siebold & Zucc.), gansui (*Euphorbia kansui* S.L.Liou ex S.B.Ho.), and jujube (*Ziziphus jujuba* Mill.). It is commonly used clinically to treat CAS and has achieved significant efficacy with few side effects (Zhao et al., 2018). Euphorbia has been widely used to treat urinary retention, malignant ascites, and edema because of its diuretic and decongestive properties. Euphorbia has been included in many formulas, such as the Ji Dai formula and Modified Shi Zao Decoction, for treating malignant ascites (Luo, 2013; He, 2015; He et al., 2017). The extracts of euphorbia have been found to have a variety of activities, such as antiviral and anti-inflammatory effects. Flos genkwa, a traditional laxative and water-removing medicine, is mainly used to treat the syndrome of fluid retained in the chest, hypochondrium, and abdominal fluid (Jiang et al., 2015). Gansuiis a dry root derived from *Euphorbia kansui* T., which is toxic and commonly used to treat pleural effusion, ascites, and edema (Zhao et al., 2019a). Euphorbia, Euphorbia kansui, and flos genkwa in SZD are toxic to the intestinal tract. Adding jujube can enhance immunity and show network analysis of the anti-inflammatory effects by regulating the gut microbiota and metabolites (Gao et al., 2023).

“Holistic regulation,” which views the organism as a whole, is what distinguishes Chinese herbal medicine. CHM practitioners focus more on the patients with diseases than on the ailments themselves, using the human body’s homeostasis as their guidance. It is challenging for a single medication to meet the needs of patients with variable and complex conditions due to the diversity of pathogenesis and the complexity of patients’ conditions. The TCM formula is derived from the wealth of rich experience in long-term clinical practice by fully utilizing the benefits of TCM differentiation and treatment. A large number of studies have confirmed the multi-target and multi-level characteristics of the mechanism of action of traditional Chinese medicine compounding, which means that modern scientific analysis should be used to study the active ingredients of compounding and the mechanism of compounding action. The characteristics of the mechanism of action of traditional Chinese medicine formulas are very similar to the completeness, systematicity, and comprehensiveness of network pharmacology, so network analysis is very suitable for the study of the pharmacological mechanism of traditional Chinese medicine formulas (Wang et al., 2022; Shang et al., 2023). There are multiple metabolites in TCM formulations. Network analysis can be used to screen potential metabolites and targets, while the potential PRM absorbed into the bloodstream at an effective concentration is a prerequisite for the therapeutic effects of TCM formulation. Therefore, pharmacokinetics revealed the exposure levels of the absorbed RPM from SZD (Zhao et al., 2021). Metabolic markers and pathways that may predict drug responses can be identified by metabolomics (Pang et al., 2019). Subsequently, integrating network analysis, pharmacokinetics, and metabolomics is a practical method to predict pharmacodynamic PRM and explore the effects of PRM.

To our knowledge, pharmacokinetic studies of CAS have never been reported before. In this paper, the metabolites and targets of SZD were screened by network analysis, and pharmacokinetic studies of the PRM of SZD were performed to explore the exposure levels in the blood. Comprehensive metabolomic studies of CAS in serum and urine were used to identify potential biomarkers of CAS. Finally, we explored the possible mechanisms of SZD for treating CAS by integrating pharmacokinetics and metabolomics. These findings contribute to the development of new therapeutic targets and treatments for CAS.

## 2 Methods

### 2.1 Reagents and chemical

Euphorbia (root, Lot: 20210324, 20210515), flos genkwa (bud, Lot: 20210427, 20210509), gansui (root tuber, Lot: 20210612, 20210718) and jujube (fruit, Lot: 20210728) were all purchased from Nanjing Tongrentang Pharmaceutical Co., Ltd. (Nanjing, China). The plant name has been checked with http://www.worldfloraonline.org. (Note: website accessed on 27 August 2023). Moreover, it was identified by Prof. Lina Guo (School of Traditional Chinese Materia Medica, Qiqihar Medical University, Qiqihar, China) as the dried root of *Euphorbia pekinensis* Rupr., the dried flower bud of *D. genkwa* Siebold & Zucc., the dried tuberous root of *Euphorbia kansui* Liou ex S.B.Ho., and the ripe fruit of *Z. jujuba* Mill., respectively, and placed in the herbarium of Chinese herbs of Qiqihar Medical University. Preparation of SZD: Euphorbia and gansui were removed from the non-medicinal parts, impurities, and foreign matter, and flos genkwa was removed from the impurities to produce the raw drinkable tablets of each herb. Pure flos genkwa was placed in a hot pan, heated with mild fire, and stir-fried until it changed color, removed, and allowed to cool, resulting in boiled flos genkwa. The above tablets were pulverized and then passed through a 200-mesh sieve. 3 g of euphorbia, gansui, and flos genkwa were accurately weighed, and 3 g of jujube was removed from the pit and weighed. The mixture was boiled in two batches and concentrated to 500 mL. Finally, SZD was obtained. The extraction rate of the extract was 31%.

Colchicine tablets (Lot: 120994) were produced by Kunming Pharmaceutical Group Co., Ltd. (Kunming, China). Colchicine tablets (Lot: 120994, packing specification of 0.5 mg × 100) were produced by Kunming Pharmaceutical Group Co., Ltd. (Kunming, China). The reference substances were supplied by Xinyang Zhongjian Quantitative Biotechnology Co., Ltd. (Henan, China), including PA (protocatechuic acid, Lot: 200406), CHA (chlorogenic acid, Lot no: 191110), CA (caffeic acid, Lot: 190503), rutin (Lot no: 200404), isovitexin (Lot: 191218), galuteolin (Lot no: 190721), UL (Umbelliferone, Lot: 190621), Luteolin (Lot: 190711), Naringenin (Lot no: 191224), Apigenin (Lot: 200824), Daphnoretin (Lot: 200516), Genkwanin (Lot no: 200518), and Schisandrin (Lot: 21022452) (internal standard, IS). The purity of all reference substances was >98%. Methanol and acetonitrile (Lot: 348604, 142085) were from high-performance liquid chromatography (HPLC, Fairfield Reagent Co., Ltd.). Formic acid (chromatographically pure, Lot: 095224) was purchased from the American Dikma Corporation.

### 2.2 Animals and groups

#### 2.2.1 Animals

Sprague Dawley rats (220 ± 20 g) of half gender were supplied by the Animal Experimental Center of Qiqihar Medical University and Beijing Weitong Lihua Laboratory Animal Co., Ltd., respectively (animal license No. SCXK (Beijing) 2007-0001, SYXK (Hei) 2021-013). Rats had free access to water. Animal room temperature: 20–22°C, humidity: 40%–70%. All protocols for animal studies were approved by the Animal Ethics Committee of Qiqihar Medical University and conducted according to the guidelines (approval number: QMU-AECC-2022-13; time: 3/6/2022).

#### 2.2.2 Grouping of rats and establishment of the CAS model

The rats were randomly divided into different groups. For pharmacokinetics, the blank group can test the comprehensive impact of factors such as instrument noise, reagents, environment, and contamination during operation on sample determination. Meanwhile, the blank experiment can reduce the experimental error, which is essential to improving the accuracy of the test results. The experimental group was given 2 mL of SZD sample solution (5 mg/kg), and the same amount of water was given to the blank group. The rats for metabolomics research were in the normal group (A), CAS model group (B) (Fortea et al., 2018), SZD treatment group (C), and colchicine treatment group (D). After the rats were acclimated in the laboratory for 1 week, the normal rats were given water, and the other groups were given 35% phenobarbital solution (first week) for 1 week of induction. Intraperitoneal injection of 10% CCL_4_ oil solution twice a week (week 2–3), the dose is 1 mL/kg, and the first dose is double; 12% CCL_4_ oil solution (week 4–5); 14% CCL_4_ oil solution (week 6–7); 16% CCL_4_ oil solution (week 8–9); 18% CCL_4_ oil solution (week 10–11); 20% CCL_4_ oil solution (week 12–14). Liver pathology was examined in 2 animals every 2–3 weeks, starting from the fourth week, to observe the dynamic changes in liver pathology during the modeling process. Colchicine solution (a mixture of colchicine tablet 13.44 mg and water 168 mL) and SZD were given to groups D and C separately from the 10th week. 1 mL/kg of drug solution was gavagely administered every time, twice a week.

10 of the 100 SD rats were chosen in the normal group, and the other 90 rats were used to establish the CAS model. The rats for pharmacodynamic research were in the same four groups as those for metabolomic experiments. During the modeling period, the body weight of the rats was closely monitored, with the abdominal circumference measured before each injection and compared with that before the last injection. The intraperitoneal injection was suspended in rats with significant body weight loss.

#### 2.2.3 Modeling standards and inspection indicators

Modeling standards (Fortea et al., 2018; Zhao et al., 2019b): ① The urine volume was significantly reduced; ② The amount of ascites increased significantly; ③ The regenerative nodules on the liver were observed with the naked eye. ④ The fibrous septa expanded and connected in liver tissue sections, and the hepatic lobules were divided to form pseudolobuli.

### 2.3 Pharmacodynamics study

#### 2.3.1 Abdominal water volume

After 14 weeks, the rats were anesthetized with ether. A 3.0 cm incision was made along the abdominal midline, and then a weighed filter paper (2.5 cm × 5.0 cm) was inserted into the abdominal cavity. After 3 min, the filter paper with fully absorbed ascites was taken out and weighed, and the difference between the two masses was calculated.

#### 2.3.2 NO and ET-1 contents and portal vein pressure

After 14 weeks of treatment, the rats in Groups A, B, C, and D were fasted and given an intraperitoneal injection of chloral hydrate (3 mL/kg) for anesthesia. After opening the abdomen, the portal vein was isolated to measure the pressure. After manometry, portal blood was collected to measure liver function and levels of vasoactive substances (NO and ET-1).

#### 2.3.3 H&E experiment

Liver tissue was taken and fixed in formalin. After dehydration with different alcohol concentrations, xylene permeabilization, and paraffin embedding, hematoxylin-eosin staining was performed, and the slide was sealed. According to animal experimental guidelines, the rats were finally euthanized.

### 2.4 Network analysis studies

Metabolites-target-pathway networks by Cytoscape 3.9.1 were constructed. Perform the following operations in sequence to draw the network: (1) The TCMSP (http://lsp.nwu.edu.cn/tcmspsearch.php) and BindingDB (https://www.bindingdb.org/bind/index.jsp) databases were used to find metabolites of SZD. The protein targets of these metabolites were predicted through the PubChem (https://pubchem.ncbi.nlm.nih.gov/) and Swiss Target Prediction (http://www.swisstargetprediction.ch/) databases. Then, the disease targets were filtered on the Gene Cards (https://www.genecards.org/) and Online Mendelian Inheritance in Man (OMIM, https://omim.org/) databases. (2) After screening the SZD-CAS crossover targets, we performed the enrichment analysis using the Cytoscape database (https://metascape.org/gp/index.html). (3) The intersection targets, drugs, metabolites, and KEGG pathways were used to establish a metabolites-targets-pathways-disease network.

### 2.5 Serum and urine sample preparation for pharmacokinetic and metabolomic analysis

Blood samples for pharmacokinetics: Approximately 500 μL of blood was collected from the ocular venous plexus at 0.08, 0.25, 0.50, 1.0, 2.0, 3.0, 4.0, 6.0, 8.0, 12.0, and 24 h after administration and placed in a centrifuge tube after rinsing with heparin. Plasma samples (200 μL) were spiked with 20 μL of schisandrin, 20 μL of hydrochloric acid solution, and 1 mL of ethyl acetate. The mixture was vortexed for 2 min and centrifuged at 12, 000 rpm for 5 min at 4°C. The supernatant was removed and blown with nitrogen at 40°C. The residue was reconstituted with 200 μL of methanol and centrifuged (12,000 rpm, 5 min) to obtain the supernatant for analysis.

Blood samples for metabolomics were placed in a 4°C non-heparinized tube. After 2 h, the serum samples were separated by centrifugation (10 min, 3,500 r min^-1^, 4°C). Add three times the amount of methanol to the serum sample (600 μL). After vortexing for 1 min, centrifugation (4°C, 12,000 rpm, 12 min) was performed. The supernatant was collected and filtered with a microporous membrane for analysis. Urine samples for metabolomic analysis (200 μL) were diluted with 800 μL of water. The mixed solution was vortexed for 1 min and then centrifuged at 12,000 rpm for 12 min. The supernatant was analyzed using the UPLC-Q-TOF/MS method. Quality control (QC) samples for validation consisted of 50 μL of mixed serum and urine samples taken separately.

### 2.6 Plasma pharmacokinetic analysis by UPLC-MS/MS

A SHIMADZU LC-30AD UPLC system and a SCIEX QTRAP 6500+ mass spectrometer were used for UPLC-MS/MS analysis. The chromatographic conditions were as follows: Waters T3 C18 column (2.1 mm × 100 mm, 1.7 μm), column temperature: 40°C; flow rate: 0.4 mL/min; injection volume: 1 µL. The mobile phases consisted of phase A (0.1% formic acid in water) and phase B (acetonitrile): 0–10 min, 95%–0% A; 10–12 min, 0%–0% A; 12–13 min, 0%–95% A; 13–16 min, 95%–95% A. The mass spectrometry conditions are: condition scan mode: MRM mode; source temperature: 550°C; electrospray voltage: 5,500 V; nebulizing gas and cone gas: 50 and 20 psi. The mass spectrometry parameters of each RPM are listed in Supplementary Table S1.

In the PK study, non-compartmental analysis was performed using Drugs and Statistics version 3.0 (DAS 3.0) software to show the results of the time of maximum plasma concentration (T_max_), the maximum concentration of the PRM (C_max_), the terminal elimination half-life (t_1/2_), and the area under the concentration-time curve (AUC).

### 2.7 Urinary and plasma metabolomic analysis by UPLC-Q-TOF/MS

The SCIEX Triple TOFR 4600 AB ACQUITY UPLC system (AB Corp., Milwaukee, WI, United States) was used in conjunction with a Micromass Quattro Micro API mass spectrometer (AB Corp., Milwaukee, WI, United States) for UPLC-Q-TOF/MS analysis. A T3 column (2.1 mm × 100 mm, 1.7 µm) was used in the UPLC analysis system. The flow rate: 0.4 mL min^-1^; injection volume: 5 μL; system temperature: 40°C; sample temperature: 4°C. A gradient elution was used, with mobile phase A (0.1% formic acid-water, FA) and phase B consisting of acetonitrile, ACN (0.1% formic acid). The gradient elution procedure is shown in Supplementary Table S2. The MS method was used to identify the obtained metabolites in serum and urine analyses. The ion source was electrospray ionization (ESI) in positive and negative detection mode; source temperature: 530°C; acquisition range: m/z 50–1,200; ion spray voltage: 4,500 V; capillary voltage: 2.5 kV (positive); 2.2 kV (negative); MS/MS collision energy: 20–50 eV; nebulizing gas flow rate: 500 L h^-1^, cone gas flow rate: 50 L h^-1^. Cone voltage: 25 V for positive mode, 40 V for negative mode.

### 2.8 Metabolomic data analysis

Micromass Marker View Applications Manager version 1.2.1 (AB Corp., Milwaukee, WI, United States) and SIMCA-P 13.0 software were used to analyze the raw data. Unsupervised principal component analysis (PCA) and orthogonal partial least-squares discriminant analysis (OPLS-DA) using SIMCA 14.1 (Umetrics AB, Umea, Sweden) were shown as the result data matrix. A joint scoring plot (S-plot) was used to find variables that contributed to differences among different groups by performing a variable significance analysis (VIP) > 1. The accuracy of the results was evaluated by parameters such as R2 and Q2. For putative identification, the mass of potential biomarkers was first determined; in particular, MS/MS information on marker fragmentation patterns was obtained. Then, we define the structure from MS/MS spectra, databases (METLIN and HMDB), and literature. The identified potential biomarkers were directed to the online website of MetaboAnalyst for metabolic pathway analysis.

### 2.9 Statistical analysis

Drug concentration-time curves and pharmacokinetic parameters were analyzed using Drug and Statistics (DAS) 3.0 software. Other data were shown as the average ±SD (‾x ± s) and analyzed by SPSS 20.0 software.

## 3 Results

### 3.1 Pharmacodynamic study results

#### 3.1.1 Performance of rats

Compared with the blank group, the rats in the model group had lusterless fur, decreased activity, weight loss, and diluted feces. Compared with the model group, the abdominal water volume was significantly reduced in the SZD and colchicine groups. (Supplementary Table S3).

#### 3.1.2 HE staining

For group A, the liver lobules were intact and clear; the liver plates were strips, with irregular hepatic sinusoids between the plates; and only a few collagen fibers were present in the confluent area and central vein. For group B, most of the typical lobular structures were destroyed; thick collagen fibrous cords were extended from the confluent area, with the central vein dividing and encircling the lobules; disorganized hepatic cords; obvious hepatocyte edema; extensive steatosis; and some necrosis; inflammatory cells infiltrated the fibrous septum. Compared with group B, the rats in the treatment groups showed significantly less structural damage to the liver lobules and narrower fibrous strips with improved hepatocyte edema; degeneration was significantly improved, and inflammatory cell infiltration was reduced (Supplementary Figure S2).

#### 3.1.3 Effect of SZD on liver function in CAS rats

The comparison of liver function levels in rats in each group is shown in Supplementary Figure S3. The levels of ALT, AST, GLB, and TBIL in group B were higher than in group A (*p* < 0.01), while the levels were downregulated after administration of SZD and colchicine (groups C and D). The ALB level in group B was lower than group A (*p* < 0.01), while the levels of ALB in groups C and D were upregulated.

Compared with the normal group, NO and ET-1 levels and portal pressure were increased in the model group. In groups C and D, NO and ET-1 levels and portal pressure decreased (Supplementary Figure S4). SZD significantly reduced the NO and ET-1 contents and decreased portal vein pressure in rats with CAS, which indicated that the SZD group showed good therapeutic effects.

### 3.2 Network analysis

**FIGURE 1:**
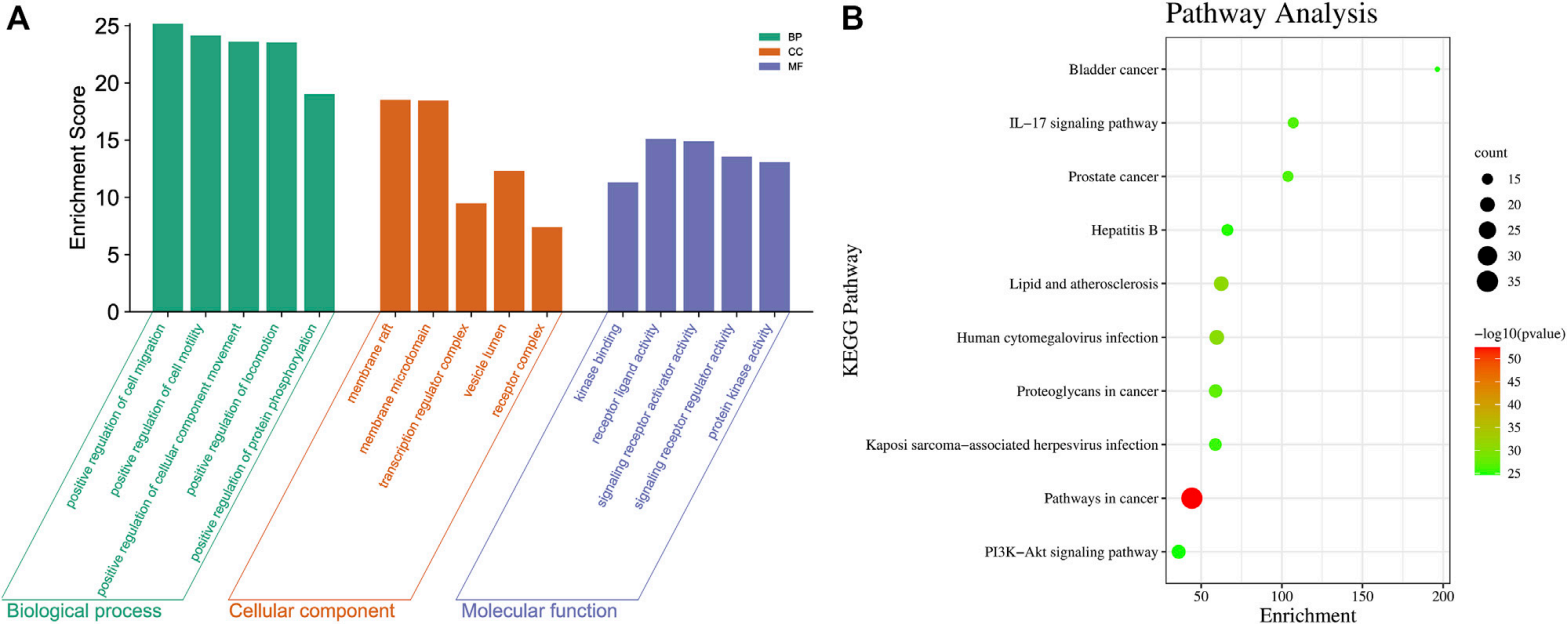
Intersection target analysis of CAS and SZD. **(A)** GO analysis chart of SZD on CAS. **(B)** KEGG pathway analysis chart of SZD on CAS.

Through the TCMSP and Binding DB databases, 74 and 800 targets of SZD and CAS were obtained, respectively. There were 45 intersecting targets between SZD and CAS that were likely to be potential targets of SZD for treating CAS. In addition, 12 metabolites related to the above intersection targets in SZD, which may be related to the treatment of CAS, were screened. To further systematically explore the mechanisms of SZD, 45 intersecting targets were uploaded into the Metascape database. The top 5 GO enrichment items are shown in Figure 1A, and the top 10 KEGG pathways are shown in Figure 1B. Among these pathways, the PI3K-Akt and hepatitis B signaling pathways may be important ways to improve CAS.

**FIGURE 2:**
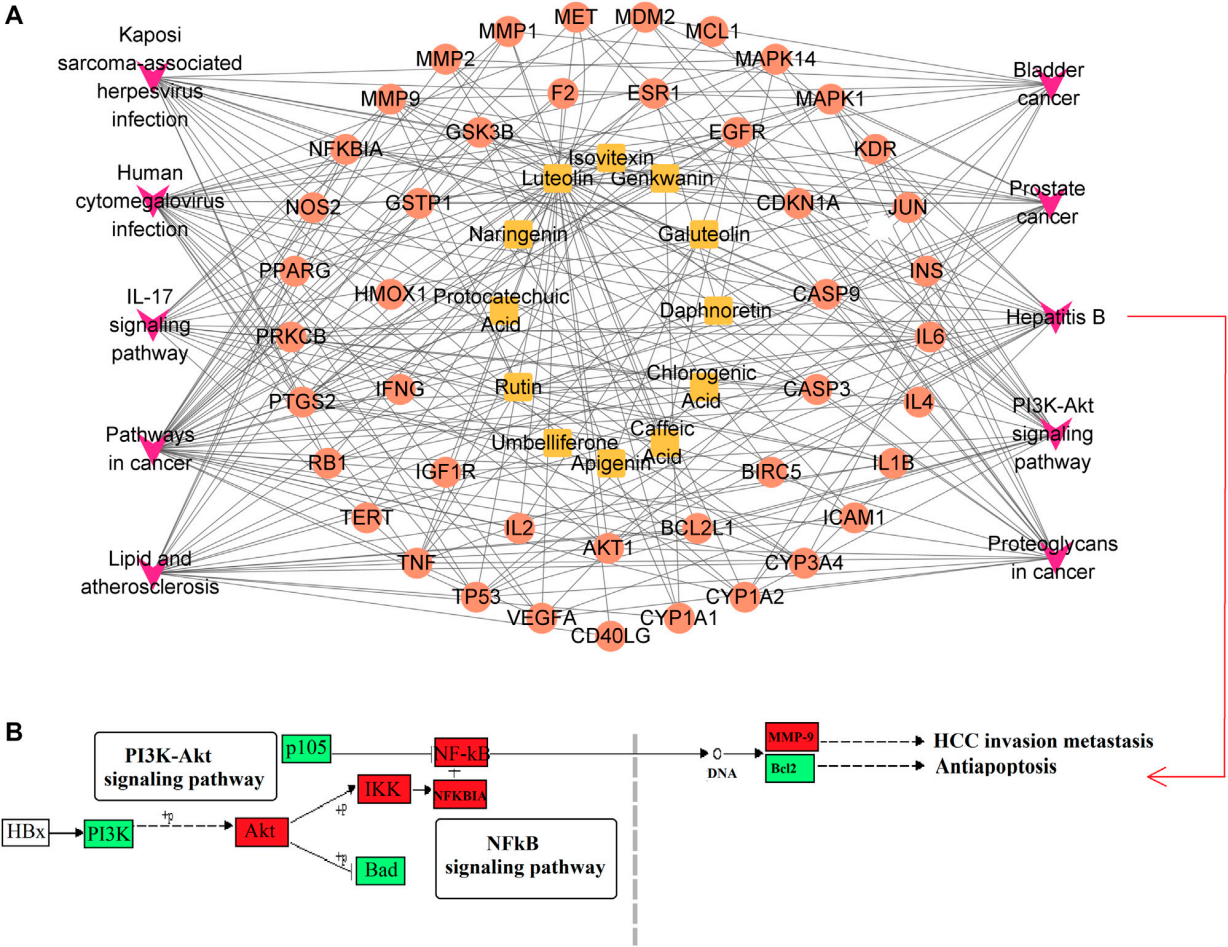
Enrichment network and pathway analysis of SZD. **(A)** Metabolites-target-pathway network. Orange square, metabolites; red circle, protein target; purple V, KEGG pathway. **(B)** Hepatitis B Signaling Pathway. Red boxes indicate targets.

A metabolite-target-pathway network was constructed to further explore the mechanism of SZD in improving CAS using the top 10 items in the enrichment pathway. The network topology analysis showed that the metabolites of SZD with the top-degree values for treating CAS were luteolin and rutin. The key targets of SZD may be TNF, CYP3A4, and CYP1A2. In the network, the targets and pathways were directly related (Figure 2A). Finally, 12 metabolites (PA, CHA, CA, rutin, isovitexin, galuteolin, UL, luteolin, naringenin, apigenin, daphnoretin, and genkwanin) were screened as the potentially active metabolites of SZD in treating CAS. As the highest-degree metabolite, luteolin acted on 40 targets (TP53, AKT1, IL6, *etc.*). MMP9 was associated with three metabolites (rutin, galuteolin, and CA). Moreover, we found that hepatitis B and PI3K-Akt, two closely linked signaling pathways, may be associated with the effects on CAS of SZD. Therefore, we separately intercepted their relationships and further analyzed them through KEGG pathway annotation. The network analysis highlighted the interactions between the PI3K-Akt signaling pathway, the hepatitis B signaling pathway, and key targets (Figure 2B).

### 3.3 Validation of the UPLC-MS/MS method

**FIGURE 3:**
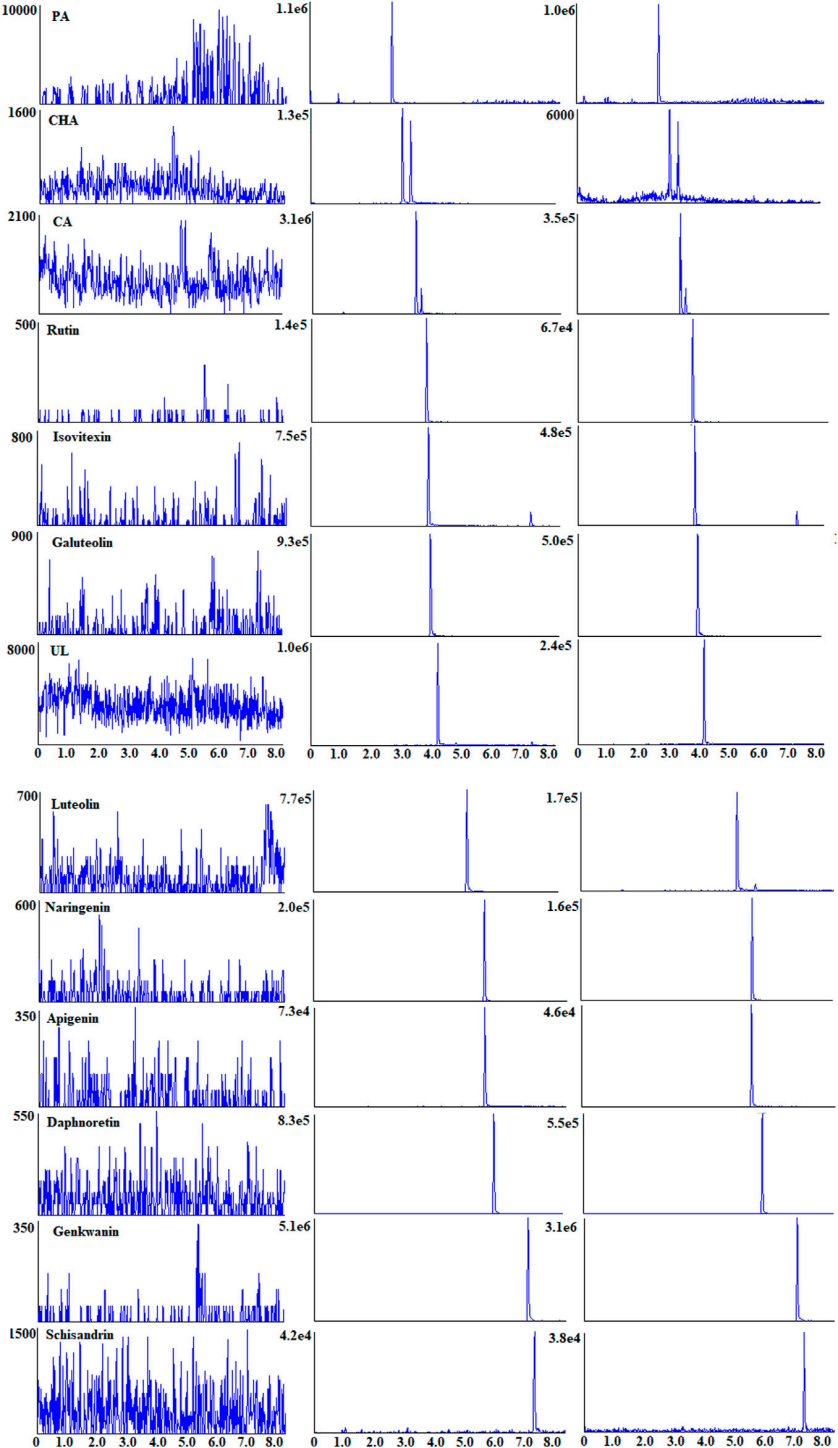
Typical MRM chromatograms of 12 analytes and IS in rat plasma.

The method was validated according to the Drugs and Biologics Method Validation Guidelines of the FDA. The validation parameters were as follows:

Specificity: The ion mass spectra of those PRM are presented in Supplementary Figure S5. The typical MRM chromatograms of 12 analytes and IS are shown in Figure 3. No endogenous species were observed within the 12 PRM and IS, indicating good specificity of the method.

Linearity and LOQ: The linear regression equations, correlation coefficients (*R* ^2^ > 0.99), linear ranges, ULOQ, and LLOQ values for the 12 PRM showed quantified calibration curves (Supplementary Table S4).

Accuracy and precision: Experimental concentrations should not deviate more than ±15% from their theoretical concentrations. The precision and accuracy results of the 12 PRM are shown in Supplementary Table S5. The within-run precision (CV) range of the 12 PRM was 1.9–11.5%. The between-run precision (CV) range was 2.8–14.9%. The results indicated that the within-run and between-run precisions, given as CV (%), did not exceed 15%. The results confirmed the excellent reproducibility and reliability in the analytical range.

Extraction recovery and matrix effect: As shown in Supplementary Table S6, the RSDs of extraction recoveries (70.3–84.2%) were less than 13.0% with consistent, accurate, and repeatable results. The matrix effects were 86.9–114.2%, and the RSDs were less than 15.0%, which showed that the established method meets the requirements.

Stability: The stability results are shown in Supplementary Table S7, including short-term stability, post-preparation stability, freeze-thaw cycle stability, and long-term stability. None of the 12 analytes showed significant degradation (short-term stability: RSD≤11.6%, RE ≤ 11.5%; post-preparation stability: RSD≤11.6%, RE<11.3%; freeze-thaw cycles: RSD≤12.6%, RE<10.8%; long-term stability: RSD≤12.5%, RE<12.8%).

### 3.4 Pharmacokinetic analysis of blood RPM after administration of SZD

**FIGURE 4:**
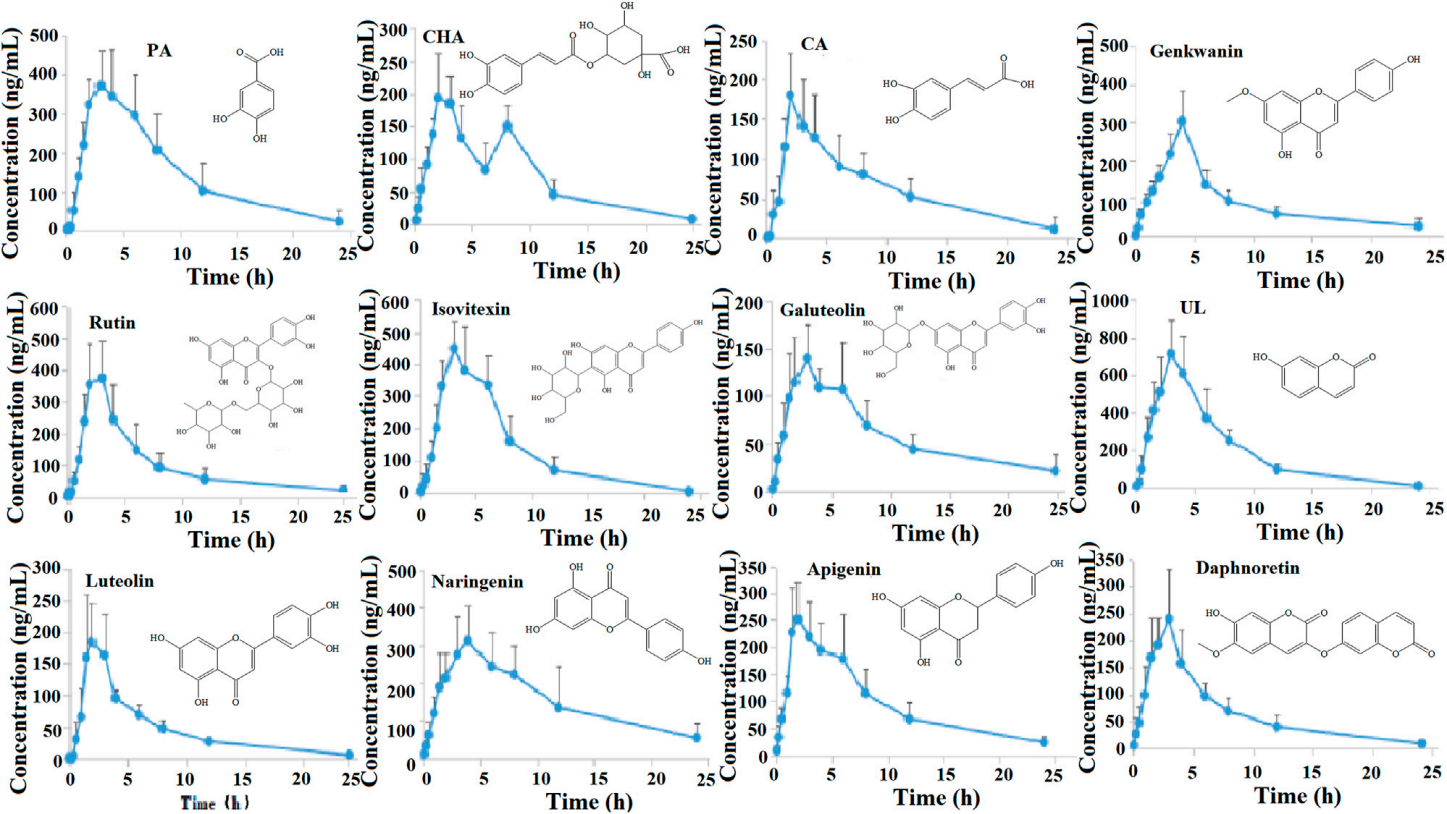
Plasma concentration-time curves of 12 PRM after oral administration of SZD.

The concentration-time curves and pharmacokinetic parameter**s** are shown in Figure 4 and Table 1, respectively. The half-life (T_1/2_) and time to peak (T_max_) of naringenin were approximately 10.78 h and 4.00 h, respectively, which were longer than the other PRM. The T_1/2_ and T_max_ of luteolin were the shortest, at approximately 4.5 h and 2.06 h, respectively; UL had a higher AUC, and the AUC_(0-∞)_ of luteolin, PA, CHA, CA, galuteolin, naringenin, and apigenin were 1,119.48 ± 193.52, 3,641.33 ± 1725.92, 1724.35 ± 570.50, 1833.80 ± 942.07, 1,492.72 ± 501.32, 4,406.82 ± 1878, and 2,492.03 ± 7 38.98 ng h/mL, respectively. Interestingly, these 12 RPM screened by network analysis were fully exposed to plasma, indicating that after intragastric administration of SZD in rats, the 12 RPM can be absorbed into the blood to exert a therapeutic effect on CAS.

**TABLE 1:** Pharmacokinetic parameters of 12 analytes in different group.

PRM	AUC_(0-t)_ (ng·h/mL)	AUC_(0-∞)_ (ng·h/mL)	T_1/2_ (h)	T_max_ (h)	C_max_ (ng/mL)
PA	3,493.30 ± 1,505.41	3,641.33 ± 1725.92	9.68 ± 2.89	3.88 ± 2.10	347.09 ± 113.56
CHA	1,680.19 ± 564.17	1724.35 ± 570.50	5.85 ± 1.41	2.25 ± 0.93	193.75 ± 59.55
CA	1,610.09 ± 558.05	1833.80 ± 942.07	6.20 ± 3.19	2.19 ± 1.30	180.30 ± 89.43
Rutin	2,384.35 ± 1,086.08	2,568.17 ± 1,380.14	5.35 ± 2.78	2.44 ± 0.62	374.49 ± 122.35
Isovitexin	3,170.12 ± 1,121.18	3,191.18 ± 1,111.83	7.31 ± 1.31	3.50 ± 1.07	448.58 ± 202.57
Galuteolin	1,379.81 ± 463.96	1,492.72 ± 501.32	6.41 ± 3.26	3.13 ± 1.53	139.52 ± 63.34
UL	4,629.52 ± 1,187.68	4,661.42 ± 1,185.75	6.34 ± 0.89	3.19 ± 0.84	710.60 ± 351.36
Luteolin	1,087.29 ± 188.46	1,119.48 ± 193.52	4.5 ± 2.61	2.06 ± 0.62	184.34 ± 68.60
Naringenin	3,637.69 ± 1,667.01	4,406.82 ± 1878.00	10.78 ± 1.87	4.00 ± 1.69	311.72 ± 130.04
Apigenin	2,278.47 ± 655.45	2,492.03 ± 738.98	7.05 ± 3.39	2.04 ± 0.88	250.69 ± 53.25
Daphnoretin	1,572.20 ± 424.58	1,626.87 ± 419.31	6.17 ± 2.41	2.81 ± 0.75	240.57 ± 111.33
Genkwanin	2077.08 ± 640.03	2,314.22 ± 884.40	5.52 ± 2.39	3.88 ± 0.52	301.84 ± 130.33

### 3.5 Metabolic profiling

#### 3.5.1 Metabonomic method validation

Supplementary Figure S6 (in Supplement) shows the chromatograms of serum and urine. Selected ion chromatographic peaks were extracted separately from serum and urine (six positive and six negative ion modes) for method validation. QC samples were applied to investigate precision, repeatability, and stability. The RSDs of retention time for instrument precision, repeatability, and system stability were 0.1%–0.3%, 0.1%–0.3%, and 0.1%–0.4%, respectively, and the RSDs of peak area were 1.9%–8.6%, 3.3%–7.3%, and 3.1%–9.3%, respectively. RE values of −7.7%–6.6% and −7.7%–9.1% were used to evaluate post-preparative stability and freeze-thaw cycle stability. The results of serum and urine samples are presented in Supplementary Table S8, which shows that the reasonable and reliable method is suitable for metabolomic analysis.

#### 3.5.2 Liver metabolic profile and potential biomarkers

The PCA score chart showed that the normal group, CAS model group, colchicine positive group, and SZD group had been separated because of the significant differences in the metabolic situation. The result showed that the metabolic environment in rats had changed in the four groups after drug administration (Supplementary Figure S7). The SZD administration group was different from the model group. It closed to normal, indicating that the metabolic situation shifted to normal after SZD treatment and that SZD could regulate the metabolic disorder in rats with CAS, consistent with the results of pharmacodynamic studies. The liver structure (Supplementary Figure S2) of Group A was intact, Group B was severely damaged, the damaged liver in Groups C and D was significantly improved, and the inflammatory cell infiltration was also significantly reduced. The concentrated quality control points indicated the good stability of the instrument.

**FIGURE 5:**
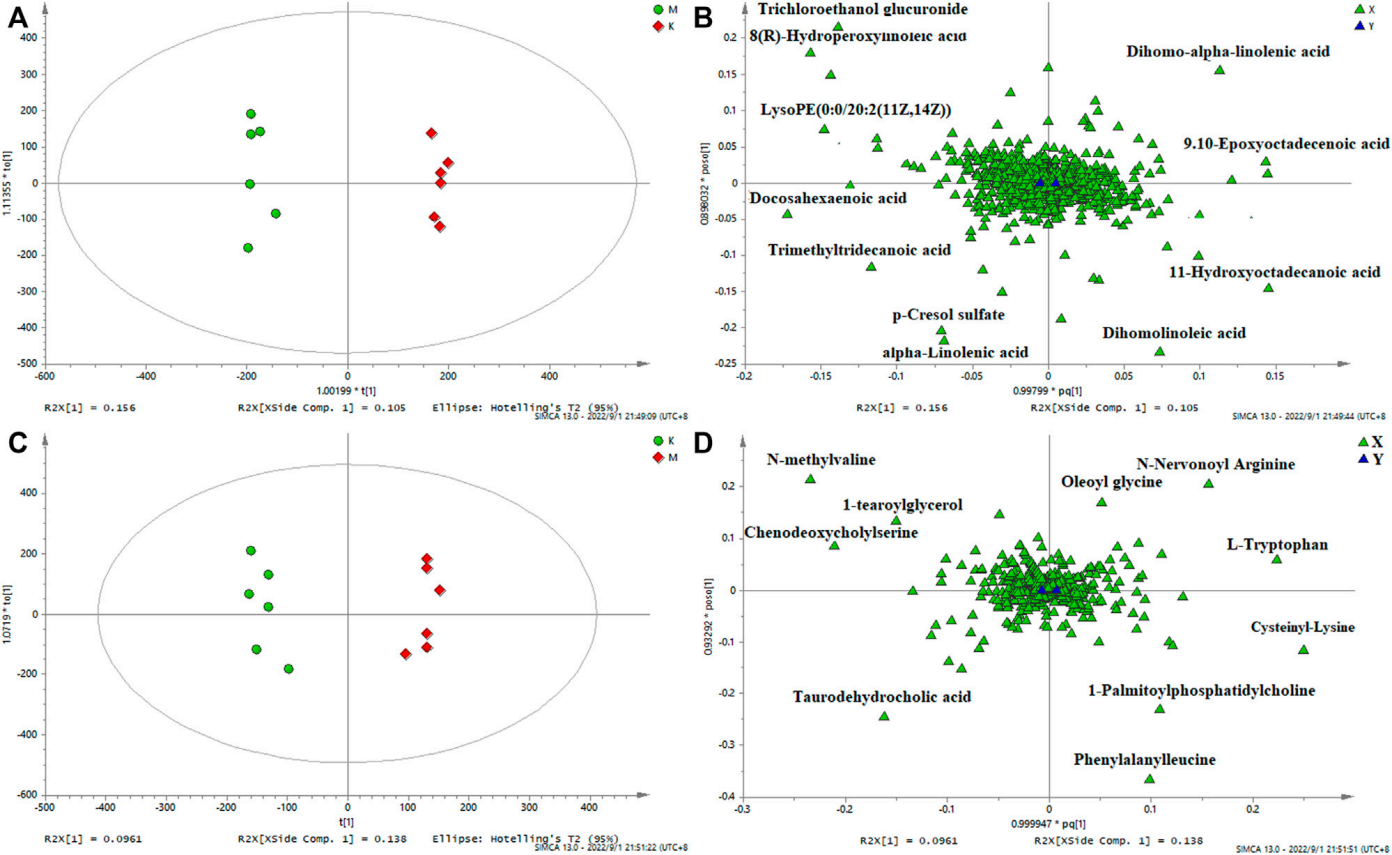
OPLS-DA serum score plots **(A)** and loading plots **(B)** in negative mode, serum score plots **(C)** and loading plots **(D)** in positive mode. (k) Control group. (m) Model group.

**FIGURE 6:**
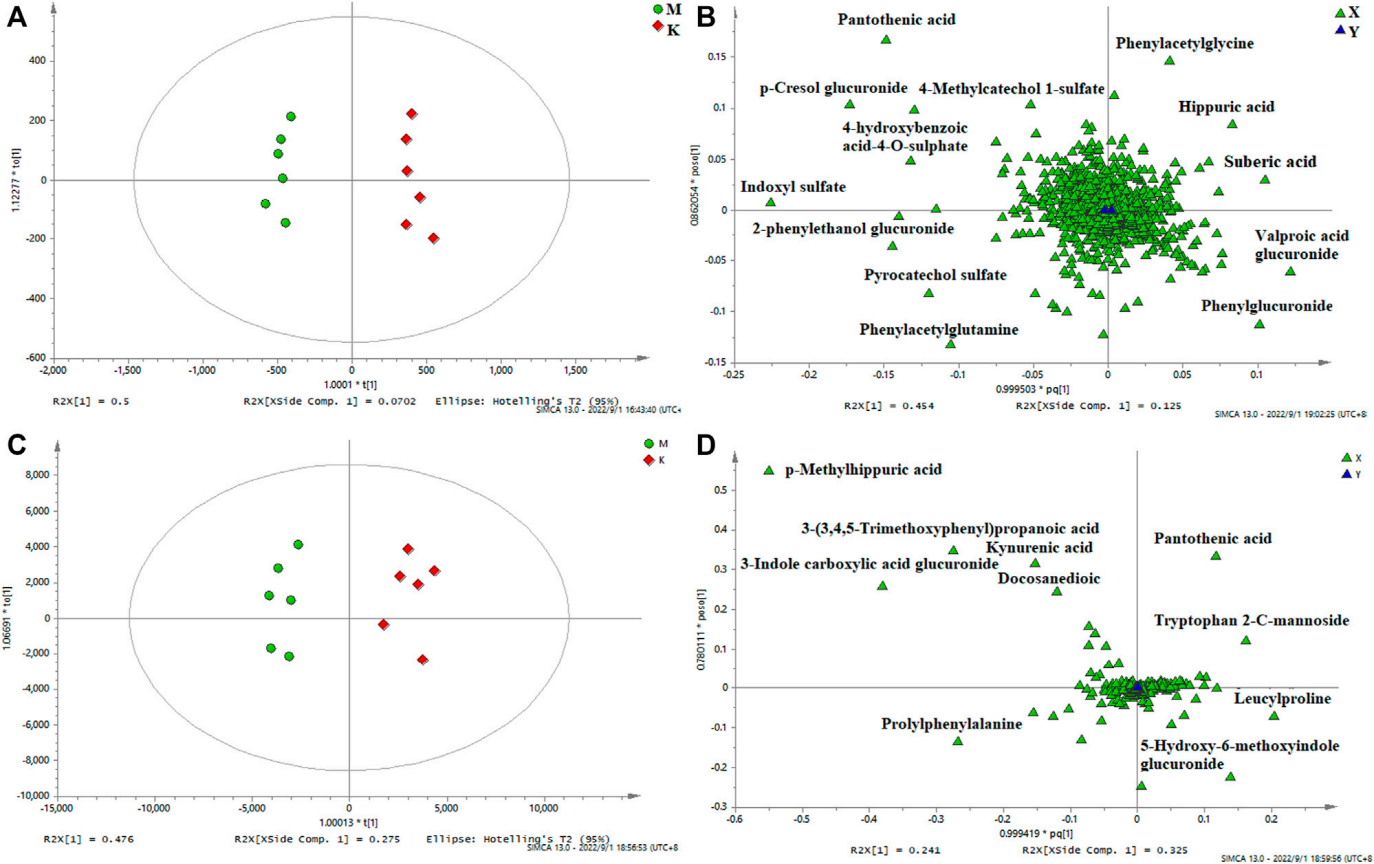
OPLS-DA urine score plots **(A)** and loading plots **(B)** in negative mode, serum score plots **(C)** and loading plots **(D)** in positive mode. (k) Control group. (m) Model group.

In order to identify the potential biomarkers, supervised OPLS-DA analysis was used to analyze significantly different metabolites in different groups, and S-plots were used to identify variables that affected the metabolic profiles. S-plot results are shown in Figure 5 and Figure 6, which prove the successful establishment of the CAS model. It was necessary to evaluate the reliability of the model by cross-testing. The results of the parameters used to evaluate model quality are summarized in Supplementary Table S9 (in the Supplement). In general, when R2 and Q2 > 0.9, the model had excellent explanation and predictive power. R2 and Q2 > 0.5 indicated that the model had good explanation and predictive ability.

**FIGURE 7:**
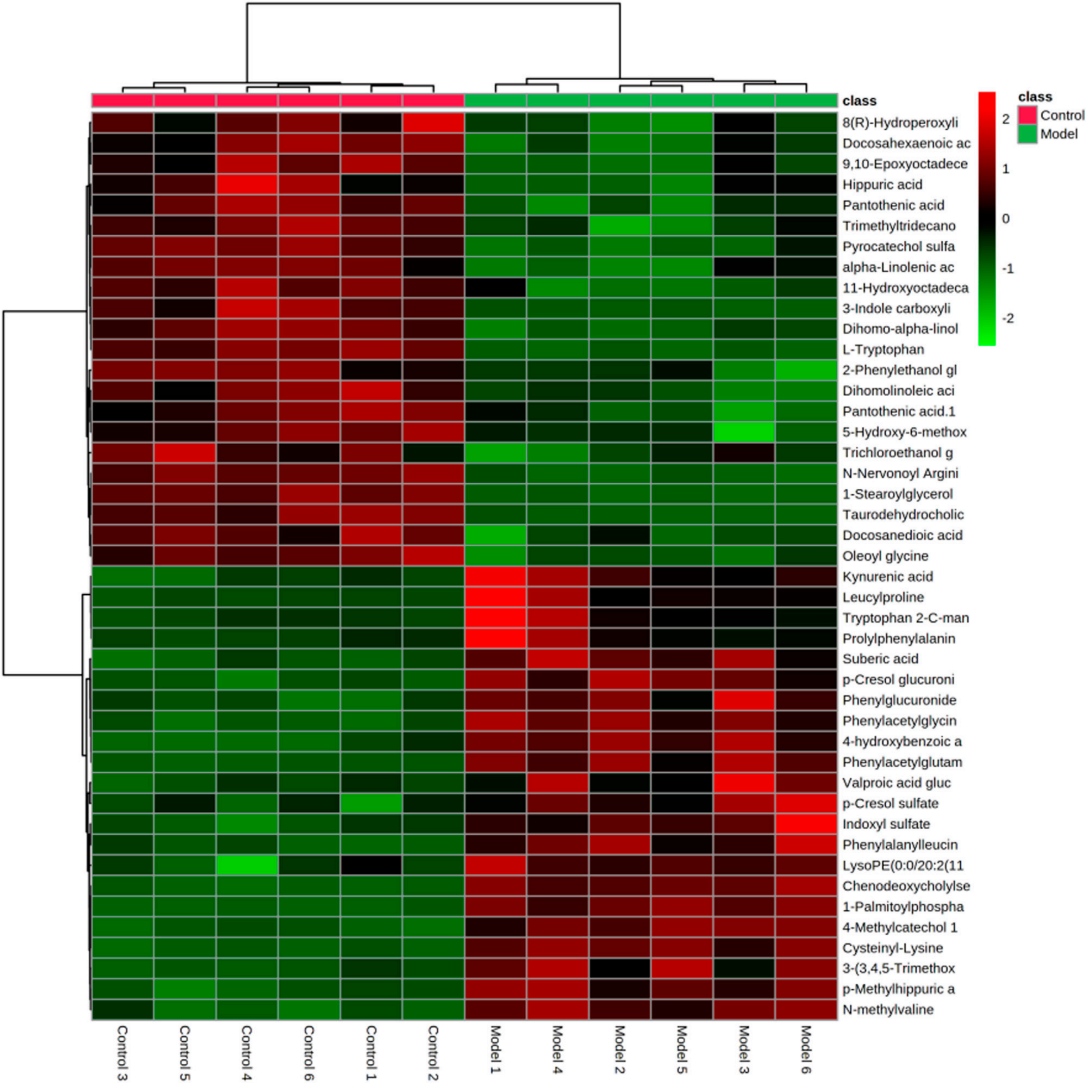
Heatmap of differential metabolite levels. With the deepening of red, the expression level of endogenous substances gradually increased, and with the deepening of green, the expression level of endogenous substances gradually decreased.

In the research process of non-targeted metabolomics, the variable importance in the projection (VIP) value was usually used as the criterion for judging potential biomarkers. By *t*-test, VIP >1, *p* < 0.05 between the normal and model groups was used as the screening condition for potential biomarkers. Difference variables were evaluated according to the mass tolerance between the obtained m/z values. Exact masses of RPM within 5 ppm (e^−6^) were used to infer metabolites. Potential biomarkers were screened and identified based on the above screening criteria and by accurately matching mass and MS/MS fragment information from the online databases HMDB (www.hmdb.ca) and METLIN (metlin.scripps.edu). Finally, 20 metabolites were screened in positive ion mode, and 24 were screened out in negative mode (Table 2). The metabolites in the serum and urine of the drug administration group were significantly different (**p < 0.01, *p < 0.05), which indicated the preventive and therapeutic effects of SZD. A heat map using MetaboAnalyst showed the metabolic patterns of 44 biomarkers (Figure 7), and the hierarchical clustering results provided a visualization of both groups.

**TABLE 2:** Potential biomarkers in serum and urine of CAS rats induced by SZD.

t_R_ (min)	m/z (Da)	Putative identification	M/C	T/M	t_R_ (min)	m/z (Da)	Putative identification	M/C	T/M
**Positive mode in rat serum sample**					**Negative mode in rat serum sample**				
2.09	132.1022	N-methylvaline	↑**	↓*	5.68	322.9486	Trichloroethanol glucuronide	↓**	↑*
4.24	205.0958	L-Tryptophan	↓**	↑**	8.8	327.2334	Docosahexaenoic acid	↓**	**↑**
6.78	250.123	Cysteinyl-Lysine or Lysylcysteine	↑**	↓**	9.01	279.2325	Dihomolinoleic acid (LA)	↓**	↑**
8.14	279.168	Phenylalanylleucine	↑**	↓**	5.83	187.0075	p-Cresol sulfate	↑**	↓**
5.69	340.2839	Oleoyl glycine	↓**	↑**	8.65	277.2182	alpha-Linolenic acid	↓**	↑*
9.94	359.3125	1-Stearoylglycerol	↓**	↑**	7.51	311.2217	8(R)-Hydroperoxylinoleic acid (LA-HPOD)	↓*	↑*
7.85	480.3332	Chenodeoxycholic acid	↑**	↓**	8.18	299.2593	11-Hydroxyoctadecanoic acid	↓**	↑**
7.91	496.3425	1-Palmitoylphosphatid-ylcholine	↑**	↓**	8.19	295.2279	9,10-Epoxyoctadecenoic acid (9, 10-EPOME)	↓**	↑*
6.02	510.254	Taurodehydrocholic acid	↑**	↓**	7.42	504.3083	LysoPE (0:0/20:2 (11Z,14Z))	↑**	↓*
7.84	523.4609	N-Nervonoyl Arginine	↑**	↓**	9.38	305.2487	Dihomo-alpha-linolenic acid	↓**	↑**
					9.45	255.2319	Trimethyltridecanoic acid	↓**	**↑**
**Positive mode in rat urine sample**						**Negative mode in rat urine sample**			
5.79	194.0827	p-Methylhippuric acid	↑**	↓**	4.96	212.0025	Indoxyl sulfate	↑**	↓**
5.41	338.0853	3-Indole carboxylic acid glucuronide	↓**	↑**	5.69	192.0677	Phenylacetylglycine	↑**	↓**
4.33	241.1061	3-(3,4,5-Trimethoxyphenyl)propanoic acid	↑**	↓**	5.77	283.0827	p-Cresol glucuronide	↑**	↓**
4.87	190.0517	Kynurenic acid	↑**	↓*	4.01	188.9869	Pyrocatechol sulfate	↓**	↑**
2.95	367.1512	Tryptophan 2-C-mannoside	↑*	↓*	6.04	297.0995	2-Phenylethanol glucuronide	↓**	↑*
10.68	371.3157	Docosanedioic acid	↓**	↑*	5.29	178.0518	Hippuric acid	↓**	↑*
2.38	229.1558	Leucylproline	↑**	↓*	5.87	173.0824	Suberic acid	↓**	↑**
3.56	220.118	Pantothenic acid	↓**	↑**	4.17	216.9817	4-hydroxybenzoic acid -4-O-sulphate	↑**	↓**
5.58	263.1396	Prolylphenylalanine	↑*	↓**	4.94	269.0683	Phenylglucuronide	↑*	↓*
3.09	340.1016	5-Hydroxy-6-methoxyindole glucuronide	↓**	↑*	5.65	203.0013	4-Methylcatechol 1-sulfate	↑**	↓**
					6.29	319.1373	Valproic acid glucuronide	↑**	↓*
					3.5	218.1035	Pantothenic acid	↓**	↑*
					5.4	263.1035	Phenylacetylglutamine	↑**	↓*

Note: M/C, change trend of model group vs. control group; T/M, change trend of SZD, treatment group vs. model group; * Metabolite has significant difference between two group (*p* < 0.05); ** Metabolite has significant difference between two group (*p* < 0.01).

**FIGURE 8:**
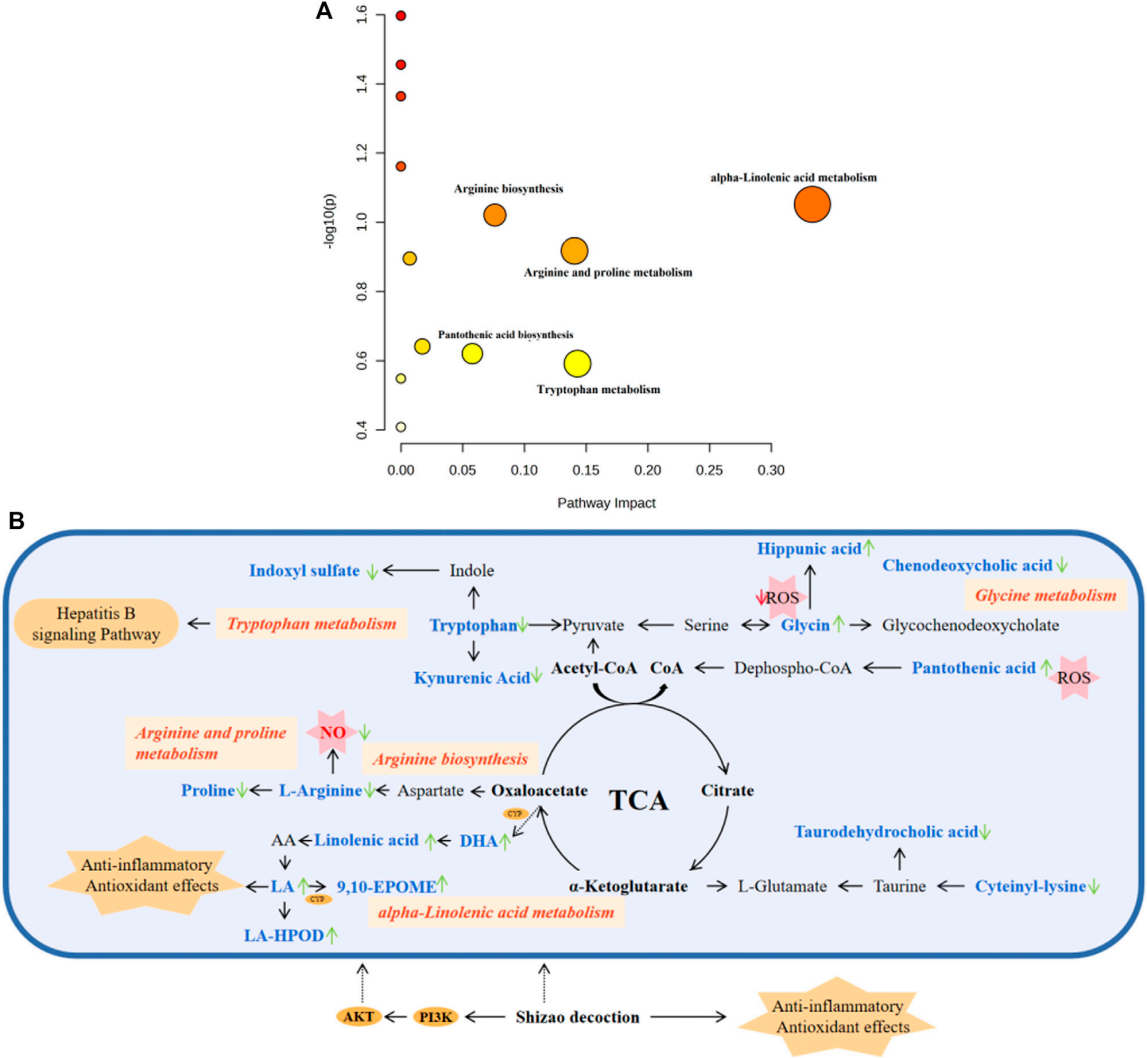
Pathway analysis of metabolites with MetaboAnalyst. **(A)** The metabolic pathways of significant metabolites in serum and urine. **(B)** Metabolic Pathway and metabolic networks analysis based on KEGG pathway database. Pink boxes, metabolic pathways; Orange oval, protein target; Orange polygon, pharmacological action. Compared to the model group, the arrows represent the change in the key metabolite levels of the SZD groups; the upward arrow indicates a rise in concentration level, while the downward arrow suggests a drop.

As shown in Figure 8A, five pathways, including tryptophan metabolism, arginine biosynthesis, alpha-linolenic acid metabolism, pantothenic acid biosynthesis, and arginine and proline metabolism, were significantly affected, among which alpha-linolenic acid metabolism had the most significant influence. The correlation between metabolites and metabolic pathways was analyzed by combining the experimental results of network analysis and metabolomics, as well as relevant literature, and the metabolic network analysis diagram is detailed in Figure 8B.

### 3.6 Comprehensive analysis of network analysis and metabolomics

**FIGURE 9:**
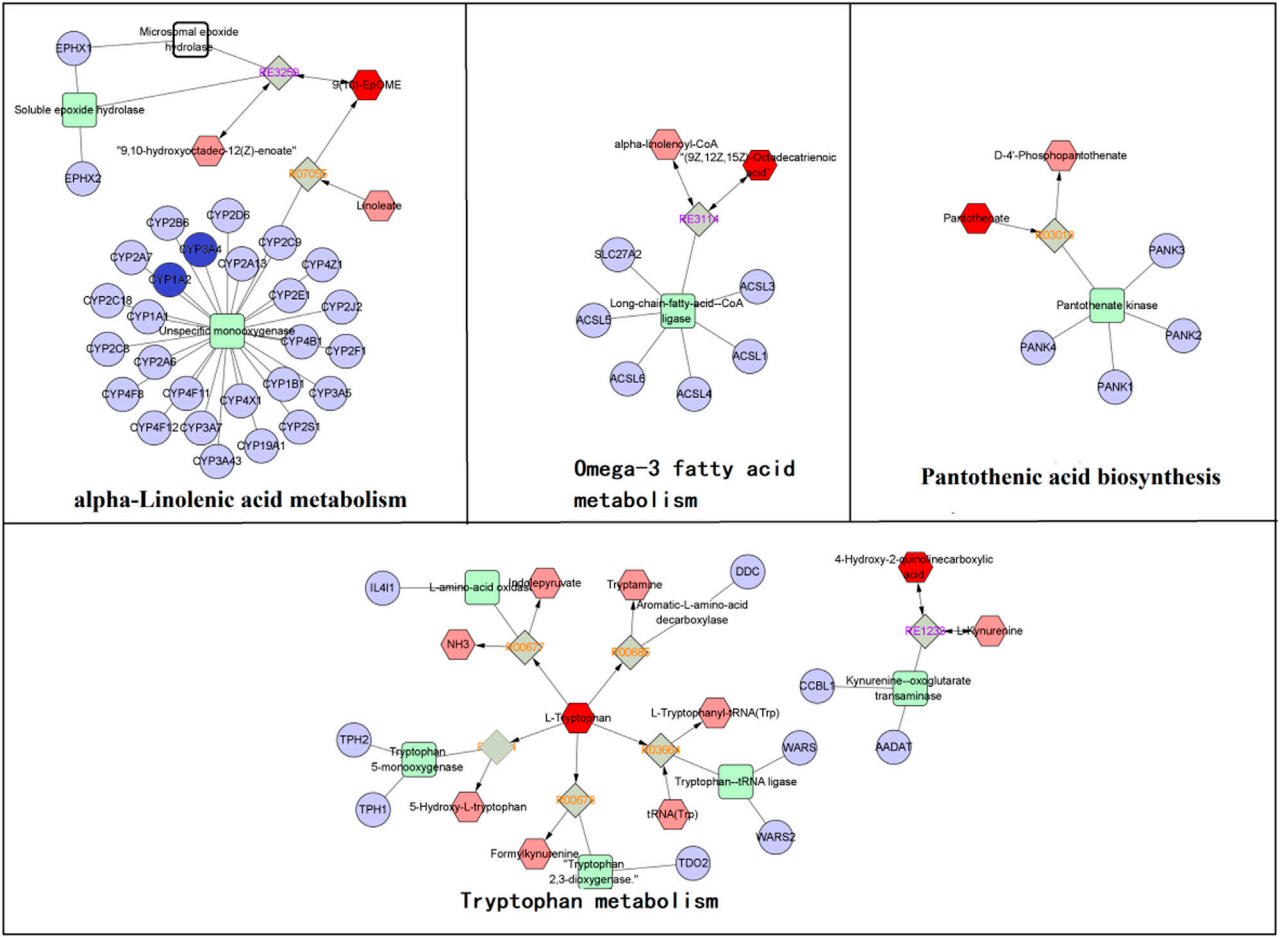
The RPM-reaction-enzyme-gene networks of the key metabolites and targets. The red hexagons, grey diamonds, green round rectangle and purple circles represent the active PRM, reactions, proteins and genes, respectively. Key metabolites, genes and pathways are marked with red boxes and red circles.

To fully understand the mechanism of SZD in treating CAS, an interaction network was constructed based on metabolomics and network analysis (Figure 9). The differential metabolites were imported into MetScape, a plug-in of Cytoscape, to obtain a PRM-targets-pathways-disease network. By matching the potential targets found in the network analysis with the genes analyzed by MetScape, two key targets (CYP3A4 and CYP1A2) were identified. The related metabolites were 9,10-epoxyoctadecenoic acid, docosahexaenoic acid (DHA), and tryptophan, which affected the following metabolic pathways: omega-3 fatty acid metabolism, linoleate metabolism, tryptophan metabolism, and pantothenic acid biosynthesis. They may be the key points in the pharmacological effect of SZD on CAS.

### 3.7 The correlation between SZD PRM and CAS-related metabolic changes

**FIGURE 10:**
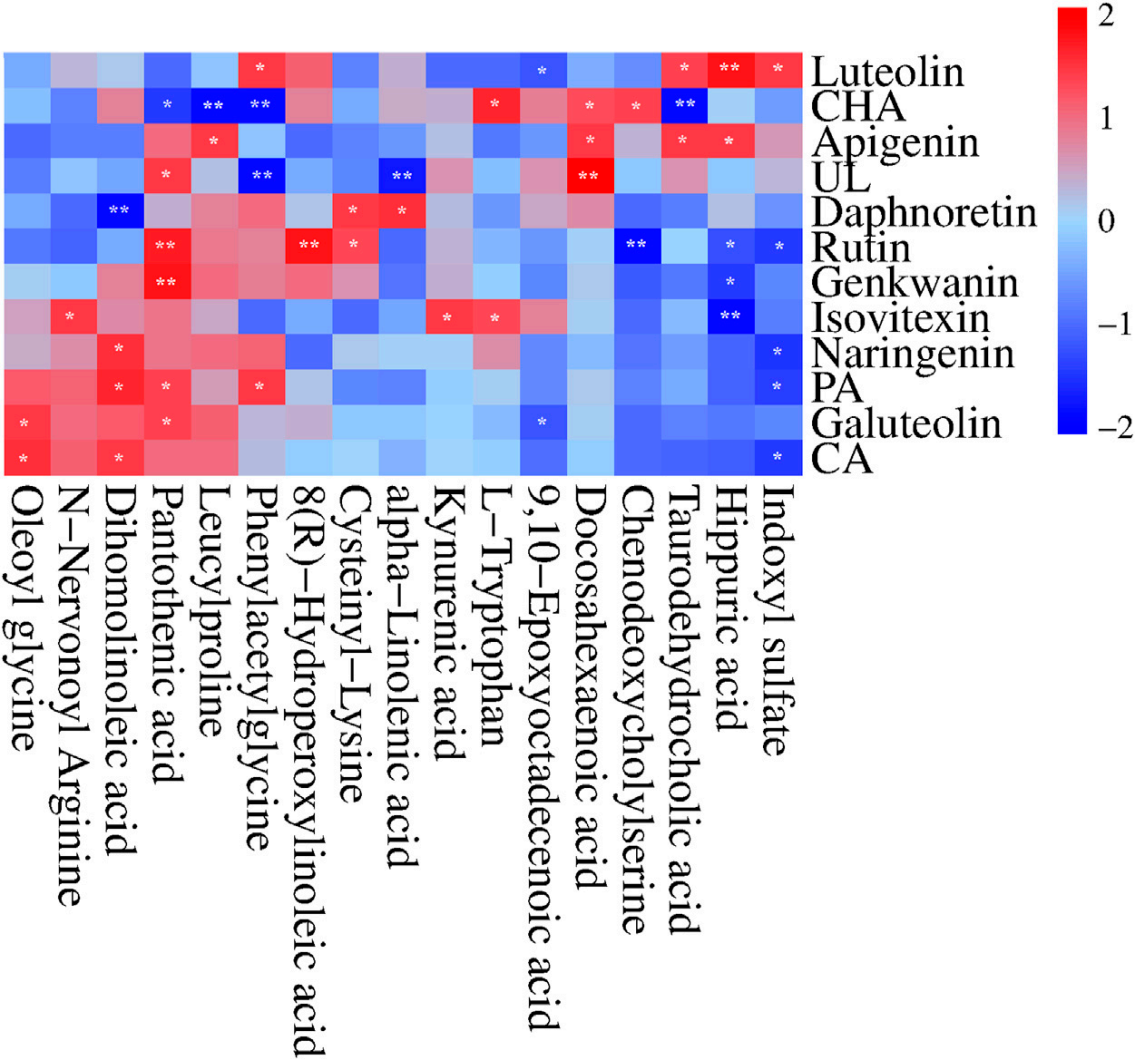
Correlation heatmap (Pearson correlation) between the 12 SZD Pharmacokinetic-markers and the plasma CAS-altered metabolites in response to SZD administration. Red and blue aquares represent the positive and negative correlations between SZD-RPM and CAS-associated endogenous metabolites, respectively. *n* = 10, *p < 0.05, **p < 0.01.

We performed Pearson correlation analysis to investigate the biological relationship between the CAS-altered endogenous metabolites and the absorbed SZD PRM (Wang et al., 2023a). Interestingly, the concentrations of the absorbed SZD PRM were significantly correlated with the 17 metabolites (|*r*| ≥ 0.7 and *p* < 0.05), with negative correlations indicated in blue and positive correlations in red (Figure 10). It was found that the RPM from SZD was mainly positively correlated with taurodehydrocholic acid, DHA, L-tryptophan, kynurenic acid, cysteinyl-lysine, 8(R)-hydroperoxylinoleic acid (LA-HPOD), pantothenic acid, dihomolinoleic acid, phenylacetylglutamine, leucylproline, and oleoyl glycine, and were negatively correlated with indoxyl sulfate, hippuric acid, chenodeoxycholic, 9,10-EPOME, alpha-linolenic acid, and N-nervonoyl arginine. According to the above correlation studies, it was shown that the 12 phytochemicals of SZD have different functions and efficacy and can synergistically resist CAS.

## 4 Discussion

The study confirmed that SZD significantly reduced liver injury and inflammatory cell infiltration in CAS rats, improved liver function, reduced ALT, AST, GLB, and TBIL levels, increased ALB levels, and also reduced NO and ET-1 levels in peripheral blood and portal vein pressure. However, systematic studies on the pharmacokinetics and metabolomics of SZD-treated CAS still need to be completed. In this study, 12 metabolites were screened through network analysis, and all have been found to have anti-CAS pharmacological effects according to relevant literature. Moreover, the pharmacokinetic parameters of the 12 potential PRM of SZD were revealed for the first time after intragastric administration. All these findings shed light on the pathogenesis of CAS and the mechanism of action of the RPM of SZD in the metabolic system of CAS.

The results of the pharmacodynamics showed that SZD could significantly attenuate liver injury and improve liver function in CAS rats. Pharmacokinetic studies indicated PA, CHA, CA, rutin, isovitexin, galuteolin, UL, luteolin, naringenin, apigenin, daphnoretin, and genkwanin have good blood exposure. Studies have shown that these 12 potential PRM of SZD have therapeutic effects on liver diseases such as hepatitis, hepatic cholestasis, cirrhosis, *etc.* While most chronic liver diseases have the pathological process of hepatitis to hepatic fibrosis to cirrhosis to hepatic carcinoma and cirrhotic ascites is one of the standard clinical manifestations of cirrhosis in its decompensated stage, these 12 potential PRM may hinder the development of cirrhotic ascites by acting on the compensatory stage of cirrhosis. So, we investigated these 12 metabolites. In addition, the metabolites with disordered expression levels were analyzed, which were mainly in the tryptophan metabolism, alpha-linolenic acid metabolism, glycerophospholipid metabolism, pantothenate and CoA biosynthesis, arginine biosynthesis and arginine, and proline metabolism.

High levels of NO production, as an important toxicity executor, play an important role in hepatotoxicity (Harstad and Klaassen, 2002; Hirunpanich et al., 2008). Arginine is a NO originator, and it can generate NO under the action of the NOS enzyme (Morris et al., 2017). To verify the effect of SZD on NO synthesis, we collected rat portal vein blood and detected the NO content. The NO level in the CAS group was significantly higher than in group A, and the NO content could be improved after SZD treatment (Supplementary Figure S4). Isovitexin can inhibit the expression of iNOS in cells and reduce NO production (Lin et al., 2005; Quintieri et al., 2008; Lucas et al., 2010). In the metabolomic study, SZD treatment led to a significant decrease in the arginine metabolite N-nervonoyl arginine. Arginine is a substrate for NO synthesis, and its downregulation can reduce NO production. Also, the changes in the levels of N-nervonoyl arginine were positively correlated with isovitexin, suggesting the mechanism of SZD in treating CAS may be involved in the L-arginine/NO pathway.

Different experimental models have shown glycine’s hepatoprotective properties (Heidari et al., 2016). Glycine decreased ROS and mitochondrial swelling (Pal et al., 2011). In addition, glycine could also restore liver mitochondrial ATP and histopathological changes. In liver cells, glycine can be conjugated with chenodeoxycholic acid to form bile salts, a primary bile acid generated in the liver from cholesterol, and elevated bile acids can cause cholestasis. After cholestasis, plasma contents of ALT, AST, TBIL, and taurocholic acid were increased, and this situation could develop into CAS over time (Zhu et al., 2018), which is the same as our pharmacodynamic study, where the levels of ALT, AST, and TBIL were higher in CAS rats than in healthy rats, and the SZD ameliorated the abnormal levels of ALT, AST, and TBIL. Studies have shown that CHA treatment reduces ALT, AST, and TBIL levels, improves liver pathological changes, and reduces cholestasis through metabolism and efflux of bile acid (Zhu et al., 2018). Apigenin can also regulate the metabolism of bile acid and improve cholestasis by reducing oxidative damage and inflammation (Zheng et al., 2021). Luteolin and rutin also have a protective effect on cholestatic liver injury (Wu et al., 2022). In addition, studies have shown that glycine can be metabolized to phenylacetylglycine, oleoyl glycine, and hippuric acid (De Simone et al., 2021). The metabolomic study in this paper found that the levels of the metabolites oleoyl glycine and hippuric acid in CAS rats were lower than those of the normal group, and phenylacetyl-glycine was higher than those of the normal rats. Phenylacetyl-glycine, oleoyl-glycine, and hippuric acid levers in CAS rats could be improved after treatment with SZD. Furthermore, after treatment with SZD, chenodeoxycholic acid and taurodehydrocholic acid in rat serum were downregulated to prevent liver damage. Also, changes in serum levels of chenodeoxycholic acid and taurodehydrocholic acid after SZD treatment were correlated with rutin, CHA, luteolin, and apigenin (Figure 10). This suggests that SZD may interfere with glycine metabolism.

Interestingly, CYP3A4, CYP1A2, and TNF may be the key targets of SZD in treating CAS through network analysis research, and DHA is closely related to these three targets. Many studies have shown that luteolin (Boeing et al., 2020), PA (Ya et al., 2021), CHA (Shi et al., 2021), CA, UL (Yin et al., 2018), galuteolin (Wang et al., 2021b), naringenin (Motallebi et al., 2022), and apigenin (Li et al., 2021a; Li et al., 2021b) have good antioxidant and anti-inflammatory effects, where CA can be used in combination with DHA to alleviate oxidative stress damage. In this study, the AUC_(0-∞)_ of luteolin, PA, CHA, CA, galuteolin, naringenin, and apigenin were 1,119.48 ± 193.52, 3,641.33 ± 1725.92, 1724.35 ± 570.50, 1833.80 ± 942.07, 1,492.72 ± 501.32, 4,406.82 ± 1878, and 2,492.03 ± 738.98 ng h/mL, respectively (Table 1). The AUC values were all larger. A larger AUC indicates greater drug absorption and increased bioavailability. Metabolomic results showed that ω-3 fatty acid metabolism (ω-3 PUFA) may be a potential metabolic pathway for SZD-treated CAS because of α-linolenic acid as the main precursor of ω-3 PUFA metabolites. Moreover, ω-3 PUFA can be metabolized to DHA by Cytochrome P450 (CYP) enzymes (Shoieb et al., 2020). In a CAS model, DHA inhibits fiber formation and prevents the development of cirrhosis by interfering with NF-κB and TGFβ signaling pathways. From the references, we know that CAS models are often induced by CCL_4_, which upregulates the inflammatory cytokine TNF, while DHA ameliorates TNF (Boyer-Diaz et al., 2020). In conclusion, there was significant DHA depletion in the livers of patients with advanced cirrhosis. Studies have shown that the development of cirrhosis can be mitigated by enhancing antioxidant capacity. Linoleic acid (LA), or dihomolinoleic acid, is the most consumed PUFA in the human diet and can enhance liver cells’ antioxidant capacity and metabolism (Chen et al., 2019; Cai et al., 2022). LA-HPOD can be metabolized into LA in the body, and LA can be metabolized into 9,10-EPOME by CYP enzymes. Enguita et al. evaluated the association between LA and its derived lipid metabolite 9,10-EpOME and biomarkers of liver injury and systemic inflammation and showed that 9,10-EpOME was reduced in patients. In addition, metabolites associated with anti-inflammation in CAS rats, including DHA, alpha-linolenic acid, LA-HPOP, LA, and 9,10-EPOME, were found to be significantly altered by metabolomics studies, and administration of SZD significantly improved the changes of these metabolites. Interestingly, positive or negative correlations were found between alpha-linolenic acid, LA, DHA, LA-HPOD, and 9,10-EPOME and eight absorbed SZD pharmacokinetics markers, including luteolin, PA, CHA, CA, UL, galuteolin, naringenin, and apigenin (Figure 10). In summary, we believe that the occurrence of CAS may be related to inflammation caused by disorders of linolenic and linoleic acid metabolism. These results indicate that SZD may increase the metabolism of linolenic acid to produce DHA by promoting CYP enzymes, thus restoring the normal level of DHA in CAS rats and achieving therapeutic effects.

Through network analysis research, it was hypothesized that hepatitis B might be a key signaling pathway for SZD to treat CAS. HBV may cause cirrhosis or even liver cancer. Besides, some experiments have demonstrated that the RPM of SZD has obvious anti-HBV effects. Through pharmacokinetic studies, the T_1/2_ and T_max_ of luteolin were the shortest, at approximately 4.5 ± 2.61 h and 2.06 ± 0.62 h, respectively, which indicates that luteolin was rapidly absorbed into the bloodstream and reached the site of action. Luteolin inhibits the production of TNF-α, IL-6, and IFN-γ in the blood and has potent anti-HBV effects (Wu et al., 2005; Wu et al., 2022). In addition, studies have shown that PRM PA, rutin, naringenin, isovitexin, CA, and CHA also exhibit anti-HBV effects (Dai et al., 2017; Jose-Abrego et al., 2022). HBV infection tends to lead to metabolic disorders, in which tryptophan metabolism can produce indoxyl sulfate and kynurenic acid (Kyn). Kyn can inhibit T-cell function and lead to T-cell apoptosis. This reaction puts the body in an immunosuppressed state and severely hinders liver function, so that tryptophan levels are consistently low in CAS patients (Jiang et al., 2020). Our experiment found that, compared with normal rats, CAS rats had higher levels of kynurenic acid and indoxyl sulfate and lower levels of tryptophan. Interestingly, SZD could regulate the levels of tryptophan, kynurenic acid, and indoxyl in CAS rats to return them to normal. Also, the contents of tryptophan, kynurenic acid, and indoxyl sulfate in serum and urine after SZD treatment were positively correlated with those of luteolin, isovitexin, and CHA, while indoxyl sulfate was negatively correlated with CA, PA, naringenin, and rutin (Figure 10). Based on the above results, it can be hypothesized that the PRM of SZD may act on the HBV signaling pathway by binding to the TNF target and interfering with the metabolism of tryptophan to exert anti-HBV effects and stop the progression of HBV to CAS.

Through network analysis, we hypothesized that the PI3K-Akt signaling pathway may play an essential role in SZD to treat CAS (Figure 1; Figure 2). In addition, many studies have shown that CHA, galuteolin (Ho et al., 2021), rutin, and genkwanin (Kim et al., 2023) can exert antioxidant effects by activating this pathway. CHA also prevents CCL_4_-induced liver fibrosis by suppressing oxidative stress in the liver and hepatic stellate cells (Shi et al., 2021). Our pharmacokinetic results revealed a significant bimodal peak after CHA absorption into the blood, possibly caused by reabsorption from the enterohepatic circulation (Xu et al., 2020). The enterohepatic circulation of CHA can prolong the half-life and maintenance time of the drug, which is generally beneficial for exerting its therapeutic effect. Through metabolomics research, we found that the content of pantothenic acid in CAS rats was lower than that in the normal group. However, SZD can improve the metabolism of pantothenic acid. Studies have shown that pantothenic acid can prevent CCL_4_-induced hepatotoxicity and oxidative stress (Eidi et al., 2012). In addition, pantothenic acid reduces oxidative stress levels mainly through the ROS-mediated PI3K-Akt signaling pathway, which may be the reason why pantothenic acid is resistant to CAS (Ya et al., 2021). Also, pantothenic acid has a positive correlation with the four active PRM of SZD, including rutin, galuteolin, and genkwanin, while CHA is negatively correlated with pantothenic acid (Figure 10). These demonstrate that SZD exerts its therapeutic effects by modulating pantothenic acid biosynthesis.

In this article, we refer to the studies in recent years that only combined network analysis and metabolomics. Our article identifies the potential RPM of SZD anti-CAS through network analysis and analyzes their migration in the bloodstream via pharmacokinetics. Additionally, an integrated network of metabolomics and network analysis was constructed using Cytoscape, ultimately hypothesizing that the key targets might be CYP3A4 and CYP1A2. This study preliminarily explored the possible mechanisms of SZD anti-CAS. In our future research, we will consider utilizing Western blot or PCR methods to validate the signaling pathways that were identified by network analysis. This would enhance the reliability of our results.

## 5 Conclusion

In this study, we first established a new integrated strategy to investigate the key targets and mechanisms of SZD in treating CAS. The results revealed that SZD can reduce liver tissue inflammation, inhibit collagen fiber hyperplasia, and improve liver function. In addition, we speculate that the effective RPM of SZD may have significant regulatory effects on the Hepatitis B signaling pathway, PI3K-Akt signaling pathway, tryptophan metabolism, arginine biosynthesis, alpha-linolenic acid metabolism, pantothenic acid biosynthesis, arginine, and proline metabolism through key targets. The data and theories provided in this study help to study the mechanism in depth and lay the foundation for clinical application.

## Data Availability

The datasets presented in this study can be found in online repositories. The names of the repository/repositories and accession number(s) can be found in the article/Supplementary Material.

## Abbreviations

ALT, Alanine aminotransferase; ALB, Albumin; AST, Aspartate transaminase; AUC, Area under curve; CA, Caffeic acid; CAS, Cirrhotic ascites; CHA, Chlorogenic acid; DHA, Docosahexaenoic acid; FA, formic acid; GLB, Globulin; RSD, Relative standard deviations; C_max_, Maximum concentration after oral dosing; SZD, Shizao decoction; LLOQ, Lower limit of quantification; OPLS-DA, Orthogonal correction partial least squares-discriminant analysis; PA, Protocatechuic acid; PCA, Principal component analysis; TBIL, Total bilirubin; 9,10-EPOME, 9,10-Epoxyoctadecenoic acid; T_1/2_, Half-life, ESI; Electrospray ionization; T_max_, Time of apparent maximum concentration measured; UPLC-Q-TOF/MS, Ultra high performance liquid chromatography coupled with quadrupole-time-of-flight mass spectrometry; IS, Internal standard; LA, Linoleic acid; LA-HPOD, 8(R)-Hydroperoxylinoleic acid; VIP, Variable significance analysis; ULOQ, Upper Limit of quantitation; UL, Umbelliferone; UPLC-MS/MS, Ultra-high Performance Liquid Chromatography tandem Mass Spectrometry.
