# Supplementary material for: Shizao decoction for cirrhotic ascites: assessing potential targets based on network analysis combined with pharmacokinetics and metabolomics
Source: Front Pharmacol. 2024 Jan 23;15:1298818. doi: 10.3389/fphar.2024.1298818 (PMC10844526; doi:10.3389/fphar.2024.1298818)
Supplement: Supplementary file 1 [file DataSheet1.docx]

**Supporting Information**


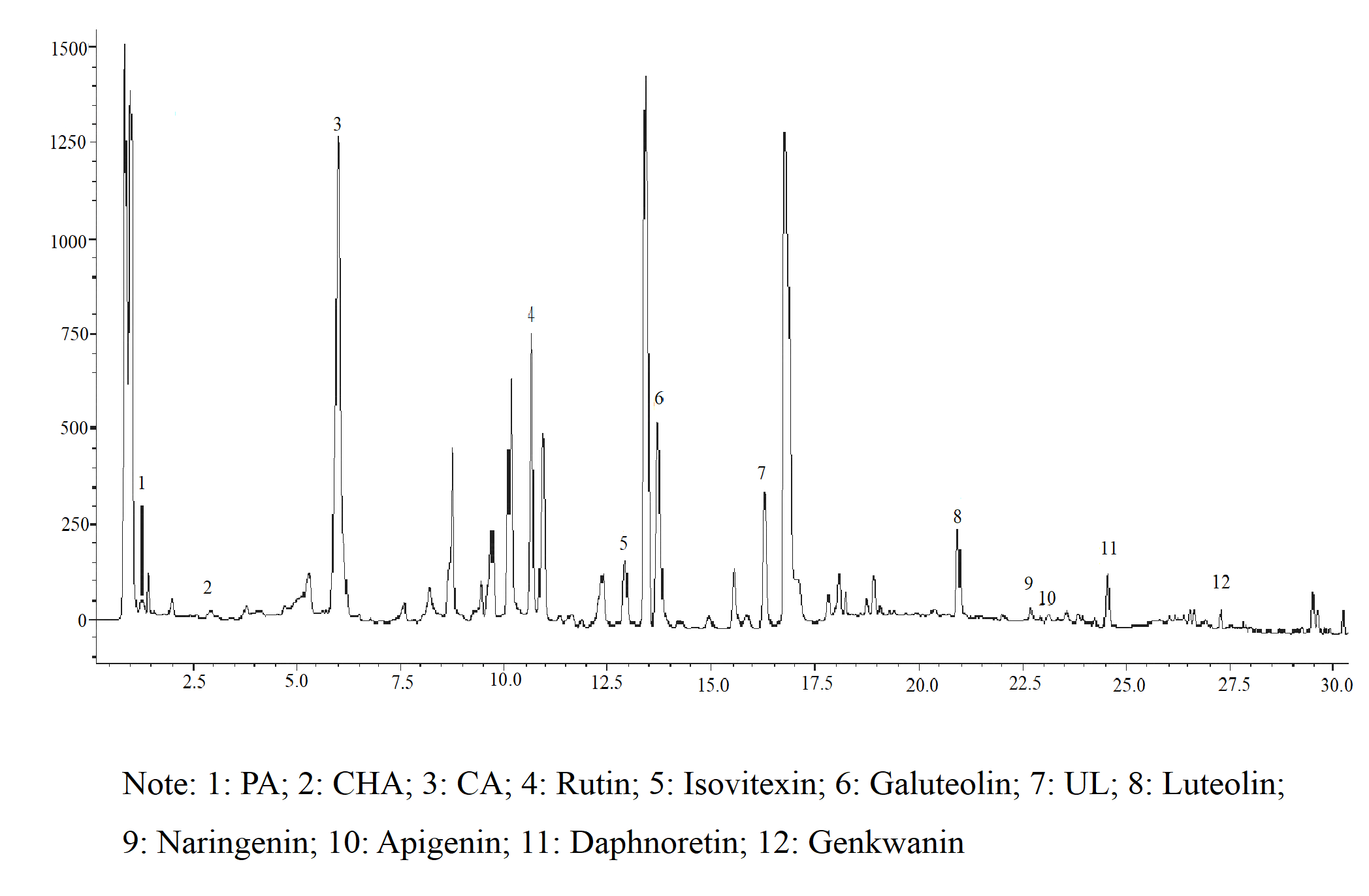


**Supplementary Figure 1.** The characteristic fingerprint of SZD extract.


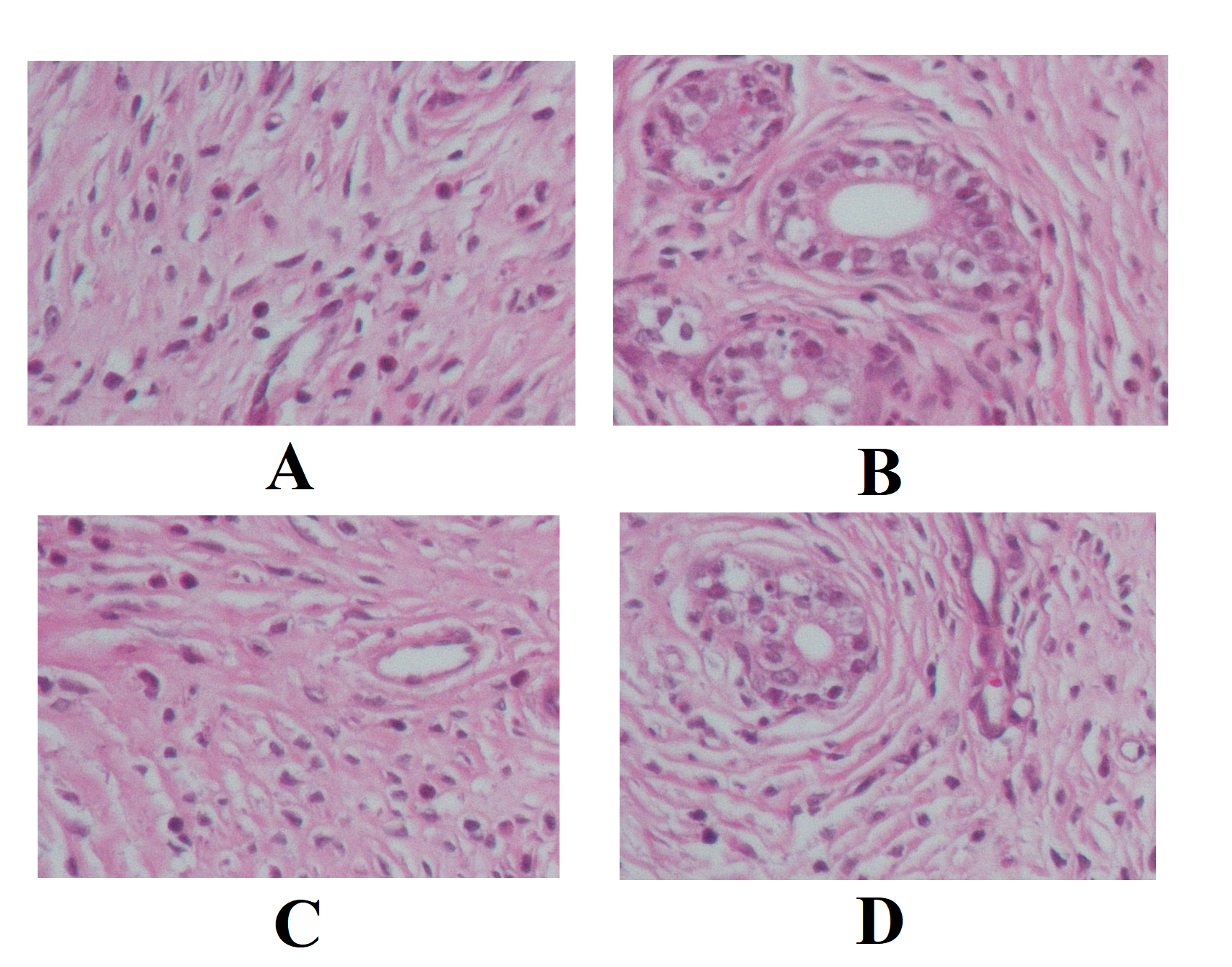


**Supplementary Figure 2.** HE staining of liver tissue (200×). Normal group **(A)**, model group **(B)**, SZD group **(C)**, colchicine group **(D)**.


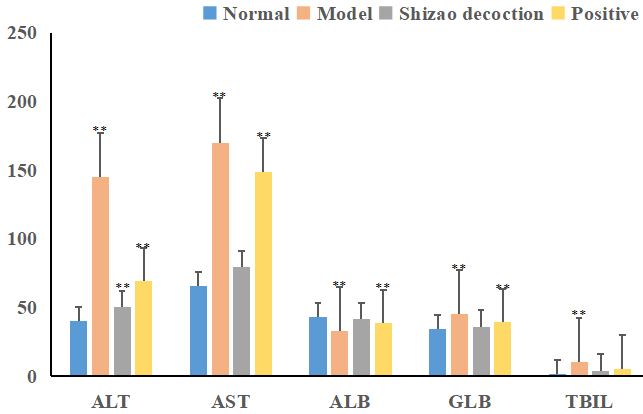


**Supplementary Figure 3.** Comparison of liver function levels of rats in each group. *n*=10, ‾x ± s, ***P*<0.01 vs. Normal.


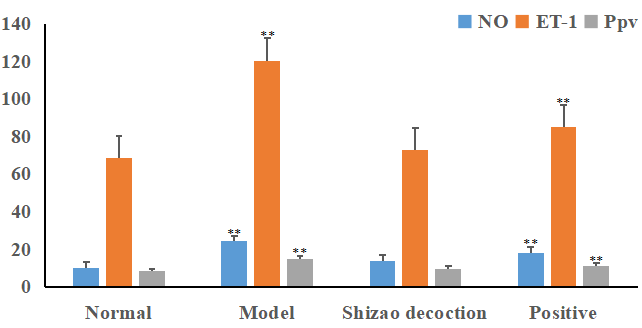


**Supplementary Figure 4.** Comparison of NO and ET-1 contents and portal vein pressure of rats in each group. *n*=10, ‾x ± s, ***P*<0.01 vs. Normal.

**
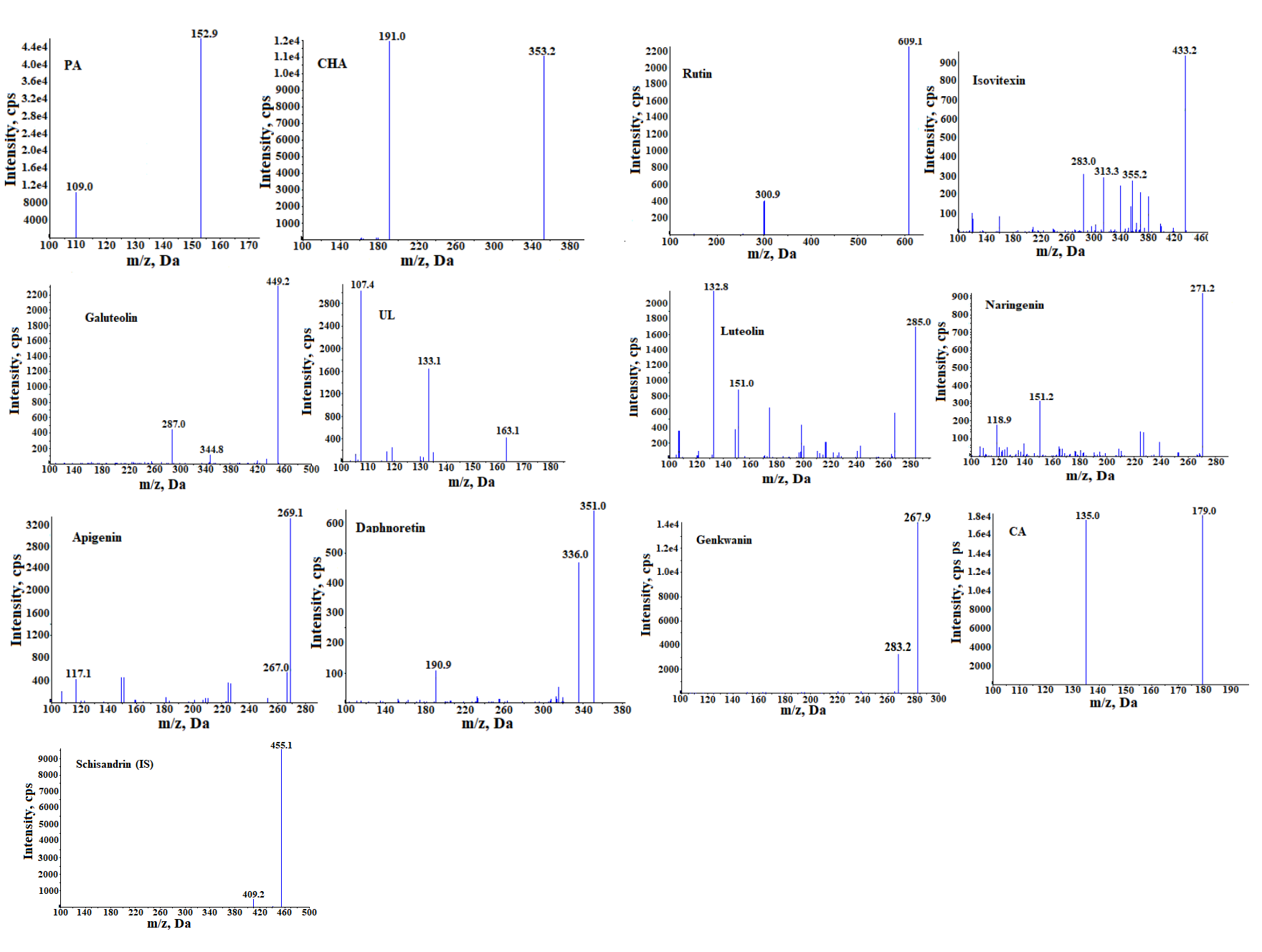
**

**Supplementary Figure 5.** The ion mass spectra of 12 analytes and IS.


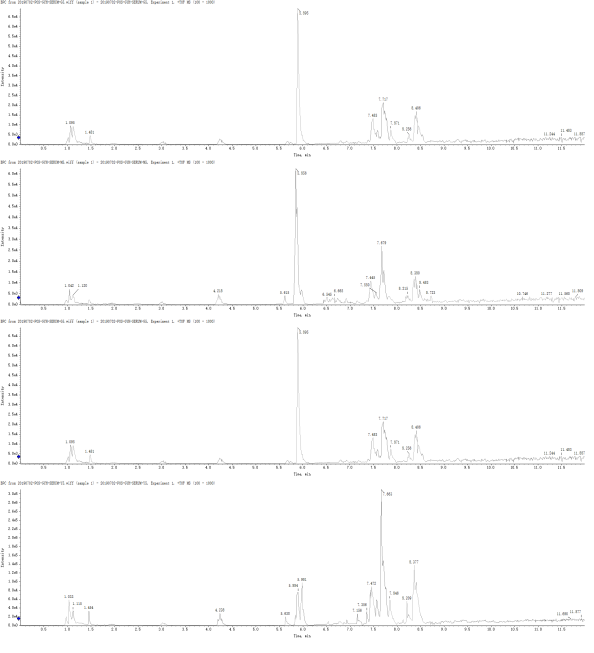


A


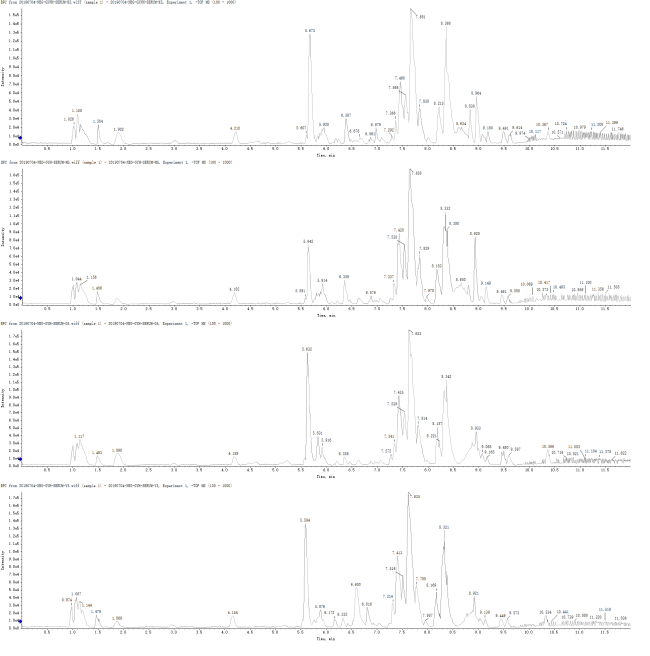


B


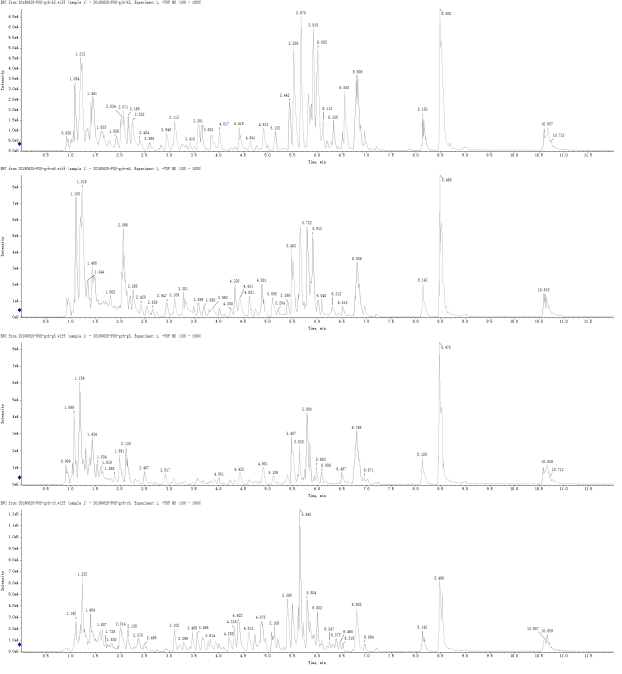


C


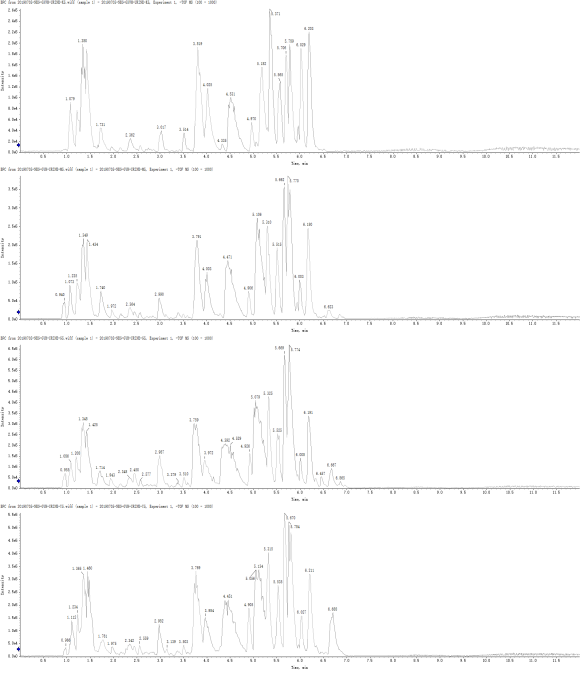


D

**Supplementary Figure 6.** Shows the chromatograms of serum and urine in positive and negative ion mode. **(A)** Serum samples in positive mode; **(B)** Serum samples in negative mode; **(C)** Urine samples in positive mode; **(D)** Urine samples in negative mode.


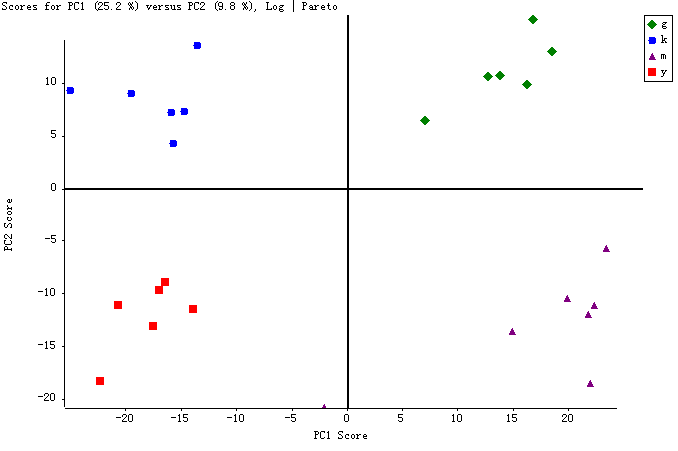


A


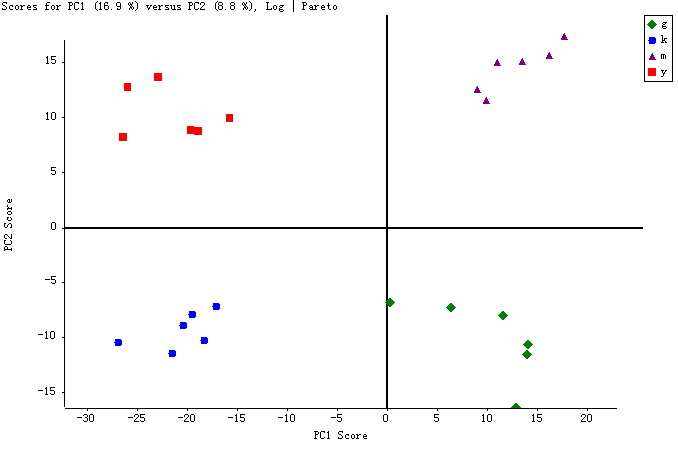


B


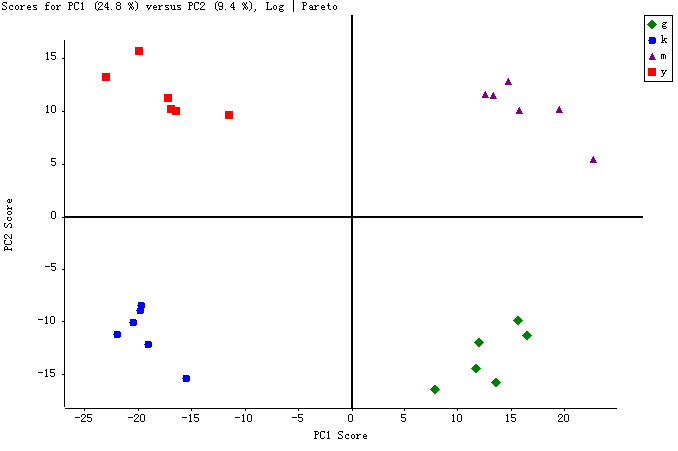


C


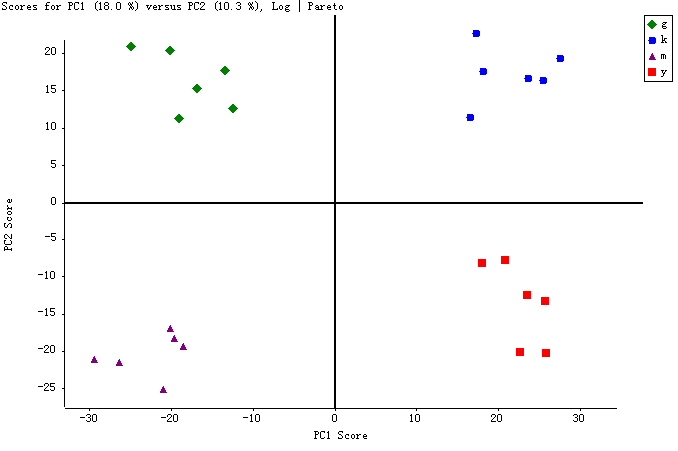


D

**Supplementary Figure 7.** PCA score plots of serum in positive mode **(A)** and negative mode **(B)**, urine in positive mode **(C)** and negative mode **(D)**. **(g)** SZD group. **(k)** Control group, **(m)** Model group. **(y)** Positive group. At the top of each PCA score plot, there are variance ratios of PC1 and PC2 represent.

**Supplementary Table 1.** Mass spectrometry parameters of 12 RPM and 1 internal standard.

| Analytes | Retention time（min） | Q1（V） | Q3（V） | DP（V） | CE （V） |
| --- | --- | --- | --- | --- | --- |
| PA | 2.78 | 152.9 | 109.0 | -48.0 | -19.0 |
| CHA | 3.11 | 353.0 | 191.0 | -27.0 | -20.0 |
| CA | 3.46 | 178.9 | 134.9 | -45.0 | -21.0 |
| Rutin | 3.84 | 609.2 | 300.1 | -160.0 | -48.0 |
| Isovitexin | 3.94 | 433.2 | 313.2 | 45.0 | 37.0 |
| Galuteolin | 4.03 | 449.2 | 287.1 | 220.0 | 22.0 |
| UL | 4.27 | 163.2 | 107.2 | 80.0 | 29.0 |
| Luteolin | 5.2 | 285.1 | 132.8 | -110.0 | -40.0 |
| Naringenin | 5.68 | 271.2 | 151.2 | -90.0 | -23.0 |
| Apigenin | 5.74 | 269.1 | 117.1 | -116.0 | -43.0 |
| Daphnoretin | 5.96 | 351.0 | 190.9 | -106.0 | -34.0 |
| Genkwanin | 7.02 | 283.2 | 267.9 | -112.0 | -32.0 |
| Schisandrin | 7.35 | 433.2 | 345.1 | 280.0 | 24.0 |

**Supplementary Table 2.** Gradient elution program for the analysis of serum and urine using ultra-performance liquid chromatography.

| Serum | | | Urine | | |
| --- | --- | --- | --- | --- | --- |
| Time  (min) | A%  (0.1%FA-  Water) | B%  (0.1%FA-  ACN) | Time  (min) | A%  (0.1%FA-  Water) | B%  (0.1%FA-  ACN) |
| 0 | 100 | 0 | 0 | 100 | 0 |
| 3 | 100 | 0 | 2 | 60 | 40 |
| 6 | 75 | 25 | 5 | 45 | 55 |
| 10 | 25 | 75 | 10 | 35 | 65 |
| 12 | 10 | 90 | 15 | 20 | 80 |

**Supplementary Table 3.** Body weight (g) per week before and after gavage and abdominal water volume (mL) at the end of 16 weeks for each group of rats($\overline{x}$±s).

| Group | Quantity | 0W | 4W | 8W | 12W | 14W | 16W | Abdominal water volume |
| --- | --- | --- | --- | --- | --- | --- | --- | --- |
| A | 20 | 245±11 | 267±4** | 295±8** | 325±9** | 337±5** | 347±7 ** | 0 |
| B | 16 | 248±6 | 254±8 | 251±7 | 254±5 | 253±6 | 248±5 | 3.9±0.5 |
| C | 18 | 250±8 | 255±6 | 259±3* | 260±6** | 262±4** | 265±7** | 0.9±0.2^Δ^ |
| D | 16 | 246±9 | 252±5 | 253±6* | 257±6** | 261±8** | 263±6** | 1.3±0.4^Δ^ |

Note: All groups compared with weight at 0 W, **p*<0.05, ***p*<0.01; abdominal water volume compared with model group, ^Δ^*p*<0.01

**Supplementary Table 4.** Linearity, LLOQs and ULOQs of 12 analytes in rat plasma.

| RPM | linear regression equations | R^2^ | linear ranges  (ng/mL) | LLOQ values  (ng/mL) | ULOQ  (ng/mL) |
| --- | --- | --- | --- | --- | --- |
| PA | Y=0.0083X-0.0025 | 0.9961 | 2-500 | 2 | 500 |
| CHA | Y=0.003X-0.001 | 0.9996 | 2-500 | 2 | 500 |
| CA | Y=0.0082X-0.0024 | 0.9984 | 1-200 | 1 | 200 |
| Rutin | Y=0.0027X-0.0072 | 0.9982 | 2-500 | 2 | 500 |
| Isovitexin | Y=0.012X+0.007 | 0.9987 | 2-500 | 2 | 500 |
| Galuteolin | Y=0.0226X-0.0089 | 0.9978 | 1-200 | 1 | 200 |
| UL | Y=0.0155X-0.0007 | 0.9975 | 5-1000 | 5 | 1000 |
| Luteolin | Y=0.0226X-0.0009 | 0.9967 | 1-200 | 1 | 200 |
| Naringenin | Y=0.004X-0.0003 | 0.9963 | 2-500 | 2 | 500 |
| Apigenin | Y=0.0019X-0.0009 | 0.999 | 2-500 | 2 | 500 |
| Daphnoretin | Y=0.0204X+0.006 | 0.9987 | 2-500 | 2 | 500 |
| Genkwanin | Y=0.1277X-0.0037 | 0.9995 | 2-500 | 2 | 500 |

**Supplementary Table 5.** Accuracy and precision of 12 analytes in rat plasma (*n*=6).

| Analytes | Theorical Concentration (ng/mL) | Within-run | | Between-run | |
| --- | --- | --- | --- | --- | --- |
|  |  | Measured concentration (ng/mL) | CV (%) | Measured concentration (ng/mL) | CV (%) |
| PA | 10 | 10.35 | 7.8 | 9.35 | 11.2 |
|  | 50 | 45.62 | 8.5 | 47.85 | 5.9 |
|  | 200 | 185.61 | 10.2 | 187.31 | 10.5 |
| CHA | 10 | 9.12 | 5.9 | 8.95 | 13.5 |
|  | 50 | 44.25 | 11.5 | 47.89 | 6.8 |
|  | 200 | 176.52 | 8.7 | 186.95 | 5.9 |
|  | 5 | 4.32 | 3.6 | 5.43 | 4.7 |
| CA | 25 | 22.56 | 6.5 | 26.58 | 7.1 |
|  | 100 | 89.65 | 2.8 | 109.64 | 3.5 |
|  | 10 | 10.68 | 1.9 | 11.23 | 14.6 |
| Rutin | 50 | 55.63 | 4.9 | 45.26 | 5.9 |
|  | 200 | 224.65 | 7.8 | 179.58 | 13.5 |
|  | 10 | 8.14 | 10.1 | 9.25 | 14.9 |
| Isovitexin | 50 | 44.68 | 6.9 | 49.25 | 11.8 |
|  | 200 | 174.63 | 8.9 | 187.62 | 7.8 |
|  | 5 | 5.43 | 9.2 | 5.46 | 8.5 |
| Galuteolin | 25 | 29.87 | 8.7 | 24.15 | 12.4 |
|  | 100 | 110.45 | 7.4 | 89.96 | 10.6 |
|  | 20 | 22.65 | 5.5 | 18.85 | 8.7 |
| UL | 100 | 88.67 | 4.9 | 88.28 | 6.9 |
|  | 500 | 436.56 | 3.8 | 469.85 | 11.5 |
|  | 5 | 4.48 | 7.5 | 4.84 | 11.7 |
| Luteolin | 25 | 26.54 | 9.1 | 24.65 | 8.7 |
|  | 100 | 108.52 | 8.8 | 88.98 | 14.6 |
|  | 10 | 8.86 | 3.7 | 8.85 | 8.2 |
| Naringenin | 50 | 44.56 | 7.1 | 52.85 | 7.1 |
|  | 200 | 184.65 | 9.4 | 216.5 | 8.9 |
|  | 10 | 9.12 | 5.9 | 11.08 | 2.8 |
| Apigenin | 50 | 44.25 | 6.1 | 54.36 | 3.5 |
|  | 200 | 177.28 | 4.8 | 181.25 | 4.9 |
|  | 10 | 8.92 | 7.5 | 8.99 | 8.7 |
| Daphnoretin | 50 | 56.35 | 5.8 | 44.58 | 10.9 |
|  | 200 | 216.5 | 10.9 | 188.55 | 8.7 |
|  | 10 | 8.92 | 8.5 | 9.52 | 4.9 |
| Genkwanin | 50 | 45.25 | 7.1 | 44.69 | 11.5 |
|  | 200 | 225.36 | 6.6 | 184.69 | 12.9 |

**Supplementary Table 6.** Extraction ecovery and matrix effect for UPLC-MS/MS analysis of analytes in rat plasma (*n*=6).

| Analytes | Nominal concentration (ng/mL) | Extraction recovery | | Matrix effect | |
| --- | --- | --- | --- | --- | --- |
|  |  | Mean (%) | RSD (%) | Mean (%) | RSD (%) |
|  | 10 | 71.5 | 6.2 | 105.6 | 8.3 |
| PA | 50 | 81.6 | 3.5 | 112.5 | 11.9 |
|  | 200 | 71.9 | 7.4 | 95.4 | 12.4 |
|  | 10 | 80.6 | 10.8 | 106.2 | 7.4 |
| CHA | 50 | 82.3 | 11.5 | 112.6 | 3.9 |
|  | 200 | 77.6 | 12.4 | 88.5 | 4.2 |
|  | 5 | 79.2 | 3.5 | 98.1 | 9.9 |
| CA | 25 | 78.4 | 6.9 | 96.5 | 10.7 |
|  | 100 | 75.3 | 8.5 | 89.4 | 8.7 |
|  | 10 | 82.1 | 7.5 | 88.7 | 5.5 |
| Daphnetin | 50 | 84.2 | 2.9 | 109.5 | 4.6 |
|  | 200 | 80.1 | 5.1 | 107.2 | 9.4 |
|  | 10 | 72.9 | 9.2 | 99.5 | 8.5 |
| Rutin | 50 | 81.5 | 6 | 94.5 | 13.6 |
|  | 200 | 70.4 | 7.8 | 93.5 | 14.1 |
|  | 10 | 72.6 | 11.6 | 92.5 | 4.9 |
| Isovitexin | 50 | 73.8 | 4.1 | 89.6 | 11.7 |
|  | 200 | 83.7 | 3.2 | 110.5 | 12.8 |
|  | 5 | 70.4 | 6.3 | 99.4 | 2.8 |
| Galuteolin | 25 | 75.9 | 5.4 | 104.6 | 13.4 |
|  | 100 | 76.4 | 9.4 | 103.8 | 10.6 |
|  | 20 | 81.4 | 2.8 | 108.7 | 7.6 |
| UL | 100 | 79.5 | 3.4 | 103.5 | 8.1 |
|  | 500 | 73.1 | 4.2 | 114.2 | 6.1 |
|  | 5 | 74.4 | 3.8 | 90.5 | 5.4 |
| Luteolin | 25 | 72.4 | 9.5 | 92.6 | 3.1 |
|  | 100 | 80.1 | 8.4 | 108.4 | 4.2 |
|  | 10 | 76.2 | 1.9 | 99.1 | 8.4 |
| Naringenin | 50 | 81.2 | 3.8 | 94.5 | 11.3 |
|  | 200 | 70.3 | 7.9 | 96.4 | 9.6 |
|  | 10 | 71.4 | 10.5 | 108.1 | 10.5 |
| Apigenin | 50 | 76.8 | 9.3 | 103.4 | 2.7 |
|  | 200 | 78.1 | 8.4 | 102.8 | 3.5 |
|  | 10 | 81.6 | 3.5 | 94.8 | 10.6 |
| Daphnoretin | 50 | 79.8 | 4.1 | 92.4 | 7.5 |
|  | 200 | 78.2 | 3.8 | 87.7 | 9.8 |
|  | 10 | 71.6 | 5.1 | 86.9 | 6.3 |
| Genkwanin | 50 | 70.8 | 9.4 | 94.9 | 8.1 |
|  | 200 | 71.9 | 6.4 | 110.7 | 7.8 |

**Supplementary Table 7.** Stability of 12 analytes under tested conditions (*n*=3).

| Analytes | Nominal concentration  (ng/mL) | Short-term (%) | | Post preparation (%) | | Freeze-thaw cycles (%) | | Long-term, (%) | |
| --- | --- | --- | --- | --- | --- | --- | --- | --- | --- |
|  |  | RE | RSD | RE | RSD | RE | RSD | RE | RSD |
|  | 10 | 10.2 | 3.5 | 6.2 | 8.5 | -11.5 | 6.3 | -12.5 | 4.7 |
| PA | 50 | -3.5 | 10.5 | -3.8 | 10.5 | 9.5 | 5.2 | 5.4 | 5.8 |
|  | 200 | -4.8 | 8.7 | 6.7 | 3.9 | -3.5 | 3.4 | 10.8 | 10.6 |
|  | 10 | 2.9 | 5.9 | -10.9 | 5.2 | -9.5 | 10.5 | 6.5 | 12.5 |
| CHA | 50 | 11.5 | 11.5 | -9.4 | 4.9 | 5.6 | 11.8 | 5.4 | 4.9 |
|  | 200 | -7.2 | 3.6 | 10.8 | 3.5 | 3.4 | 8.5 | 3.5 | 8.2 |
|  | 5 | 8.4 | 4.8 | 7.9 | 6.8 | 2.9 | 3.4 | 8.7 | 6.4 |
| CA | 25 | -10.4 | 11.6 | 3.8 | 9.1 | 10.8 | 4.9 | 9.1 | 9.1 |
|  | 100 | 6.4 | 8.1 | 2.9 | 7.7 | -10.3 | 5.8 | -10.4 | 8.2 |
|  | 10 | 9.2 | 10.4 | 8.7 | 6.6 | -11.2 | 7.2 | 4.9 | 7.6 |
| Daphnetin | 50 | 4.5 | 5.1 | -9.5 | 9.5 | 8.5 | 7.6 | 3.9 | 8.1 |
|  | 200 | -5.1 | 2.9 | 9.4 | 11.2 | -2.9 | 5.9 | 3.2 | 6.5 |
|  | 10 | -3.5 | 6.1 | -7.9 | 8.7 | -9.4 | 11.5 | 4.7 | 7.7 |
| Rutin | 50 | 11.4 | 7.2 | 6.4 | 2.9 | 8.4 | 8.4 | 6.9 | 4.6 |
|  | 200 | 6.8 | 10.1 | 5.8 | 3.8 | -5.8 | 10.9 | 9.1 | 8.7 |
|  | 10 | 3.7 | 8.1 | 11.3 | 7.2 | -10.5 | 12.6 | 10.6 | 6.9 |
| Isovitexin | 50 | -3.2 | 5.7 | -10.6 | 6.2 | 8.9 | 5.4 | 11.4 | 12.4 |
|  | 200 | 6.4 | 4.3 | -7.8 | 3.6 | 3.6 | 10.6 | -9.2 | 11.5 |
|  | 5 | 1.8 | 1.9 | -3.6 | 4.1 | 4.7 | 11.4 | -4.8 | 3.4 |
| Galuteolin | 25 | -2.7 | 5.4 | 8.9 | 5.5 | -5.9 | 8.9 | 6.7 | 4.9 |
|  | 100 | -9.8 | 3.2 | -11.4 | 6.9 | -4.9 | 5.2 | 10.5 | 8.5 |
|  | 20 | 8.1 | 4.1 | -9.1 | 10.2 | 8.5 | 3.4 | -11.6 | 6.4 |
| UL | 100 | 7.1 | 2.8 | 2.9 | 8.4 | -9.7 | 2.4 | 12.8 | 12.4 |
|  | 500 | 3.9 | 7.6 | 4.8 | 9.9 | 5.6 | 3.6 | 5.4 | 5.7 |
|  | 5 | 6.5 | 5.1 | 6.5 | 10.9 | -10.4 | 6.4 | 7.1 | 7.4 |
| Luteolin | 25 | -10.2 | 3.8 | -8.7 | 3.8 | 7.4 | 8.8 | 3.6 | 10.5 |
|  | 100 | -9.6 | 4.9 | 4.7 | 4.1 | 4.9 | 5.9 | 4.8 | 6.4 |
|  | 10 | 6.5 | 8.9 | -3.9 | 5.6 | 6.3 | 12.5 | 12.4 | 9.2 |
| Naringenin | 50 | 3.3 | 9.4 | 5.4 | 2.7 | -3.1 | 9.4 | 3.8 | 12.1 |
|  | 200 | -4.1 | 10.6 | -4.9 | 3.8 | 2.9 | 10.1 | -4.7 | 5.2 |
|  | 10 | -2.8 | 8.8 | 5.2 | 4.3 | 10.5 | 12.4 | -6.8 | 6.4 |
| Apigenin | 50 | 8.1 | 6.1 | 4.1 | 10.8 | -6.9 | 6.9 | -10.4 | 3.5 |
|  | 200 | 7.3 | 6.4 | -10.1 | 6.9 | 8.4 | 9.2 | 3.5 | 5.5 |
|  | 10 | 9.4 | 3.6 | 7.9 | 8.4 | -4.9 | 2.4 | 6.4 | 4.8 |
| Daphnoretin | 50 | -3.8 | 5.4 | 4.8 | 11.6 | -5.5 | 3.7 | 8.4 | 7.9 |
|  | 200 | -4.7 | 8.1 | -3.6 | 6.9 | 6.6 | 11.5 | -9.7 | 8.7 |
|  | 10 | 5.4 | 7.4 | -5.8 | 7.4 | 7.1 | 6.4 | 6.4 | 8.4 |
| Genkwanin | 50 | 5.9 | 5.1 | 8.4 | 8.2 | -9.2 | 9.5 | 7.1 | 6.5 |
|  | 200 | -10.3 | 3.1 | -10.5 | 6.8 | 8.8 | 11.7 | 8.5 | 6.9 |

**Supplementary Table 8.** The analytical performance of serum and urine samples.

| t_R_-m/z | Precision  (RSD, %) | | Repeatability  (RSD, %) | | System stability  (RSD, %) | | Post-preparative stability (RE, %) | Freeze-thaw cycles stability (RE, %) |
| --- | --- | --- | --- | --- | --- | --- | --- | --- |
|  | t_R_ | A | t_R_ | A | t_R_ | A | A | A |
| Serum sample in positive mode | | | | | | | | |
| 2.01-132.1058 | 0.2 | 3.5 | 0.1 | 5.9 | 0.3 | 4.8 | 6.6 | -5.7 |
| 4.22-227.0807 | 0.1 | 2.7 | 0.1 | 6.3 | 0.1 | 7.1 | -7.12 | -7.7 |
| 5.62-632.8342 | 0.2 | 2.4 | 0.2 | 4.4 | 0.4 | 5.4 | -4.4 | 7.3 |
| 5.88-167.1728 | 0.3 | 2.9 | 0.3 | 3.7 | 0.2 | 5.0 | 4.7 | -5.6 |
| 7.42-568.3633 | 0.2 | 3.3 | 0.1 | 4.6 | 0.3 | 4.7 | 5.7 | 6.5 |
| 8.40-546.3745 | 0.2 | 3.6 | 0.2 | 3.8 | 0.2 | 3.1 | 4.8 | 4.4 |
| Serum sample in negative mode | | | | | | | | |
| 4.18-204.0852 | 0.1 | 1.9 | 0.1 | 3.3 | 0.2 | 6.8 | -7.3 | 9.1 |
| 5.63-390.9364 | 0.1 | 2.6 | 0.2 | 3.8 | 0.3 | 5.5 | 6.3 | 7.8 |
| 6.37-498.288 | 0.3 | 3.3 | 0.2 | 4.1 | 0.2 | 5.9 | -5.2 | -6.4 |
| 7.42-564.3303 | 0.2 | 2.8 | 0.2 | 4.6 | 0.2 | 7.2 | -7.7 | -6.8 |
| 8.33-636.3486 | 0.2 | 3.5 | 0.1 | 3.9 | 0.3 | 8.1 | 8.1 | 7.3 |
| 9.48-922.5768 |  |  |  |  |  |  |  |  |
| Urine sample in positive mode | | | | | | | | |
| 2.08-241.1556 | 0.2 | 5.4 | 0.3 | 7.2 | 0.3 | 6.8 | 4.6 | 7.4 |
| 3.09- 340.1016 | 0.1 | 6.1 | 0.3 | 5.6 | 0.3 | 5.6 | -3.4 | -6.3 |
| 5.13-372.2387 | 0.1 | 4.6 | 0.2 | 4.8 | 0.2 | 4.0 | -6.8 | 5.3 |
| 5.95-588.4063 | 0.2 | 5.7 | 0.2 | 4.5 | 0.3 | 7.9 | -5.1 | -4.4 |
| 6.07-475.249 | 0.2 | 3.2 | 0.1 | 6.9 | 0.2 | 6.1 | 4.7 | 3.7 |
| 8.49-786.5954 |  |  |  |  |  |  |  |  |
| Urine sample in negative mode | | | | | | | | |
| 2.31-215.104 | 0.2 | 4.2 | 0.2 | 3.8 | 0.2 | 5.2 | 6.8 | 2.8 |
| 3.67-239.9969 | 0.2 | 2.8 | 0.3 | 5.9 | 0.4 | 4.6 | -5.6 | 3.3 |
| 4.43-368.971 | 0.1 | 3.3 | 0.2 | 6.1 | 0.2 | 8.8 | -4.9 | -5.2 |
| 5.11-462.9681 | 0.2 | 6.4 | 0.2 | 7.3 | 0.3 | 4.1 | 3.7 | -5.7 |
| 5.68-622.1759 | 0.3 | 3.6 | 0.1 | 4.6 | 0.3 | 9.3 | 5.2 | 4.4 |
| 6.19-425.0335 | 0.2 | 5.5 | 0.3 | 5.2 | 0.3 | 5.7 | 6.1 | 7.2 |

Note: RSD, Relative standard deviation; RE, Relative error; A, Peak area.

**Supplementary Table 9.** Summary of parameters for evaluating model quality. P (positive mode), N (negative mode).

| Normal vs Model | OPLS-DA | | | |
| --- | --- | --- | --- | --- |
|  | Mode | R2X | R2Y | Q2 |
| Serum sample | P | 0.592 | 0.983 | 0.805 |
|  | N | 0.734 | 0.999 | 0.885 |
| Urine sample | P | 0.323 | 0.943 | 0.702 |
|  | N | 0.572 | 0.997 | 0.979 |
